# Morbidity-bridging metabolic pathways: linking early cardiovascular disease risk and depression symptoms using a multi-modal approach

**DOI:** 10.1093/ehjopen/oeaf038

**Published:** 2025-04-16

**Authors:** Angela Koloi, Arja Rydin, Yuri Milaneschi, Femke Lamers, Jos A Bosch, Emma Pruin, Sander W van der Laan, Pashupati P Mishra, Terho Lehtimäki, Mika Kähönen, Olli T Raitakari, Dimitrios I Fotiadis, Rick Quax

**Affiliations:** Unit of Medical Technology and Intelligent Information Systems, Department of Materials Science and Engineering, University of Ioannina, Ioannina, Greece; Department of Biological Applications and Technology, University of Ioannina, Ioannina, Greece; Department of Clinical Psychology, University of Amsterdam, Amsterdam, The Netherlands; Department of Psychiatry, Amsterdam UMC Location Vrije Universiteit Amsterdam, Boelelaan, Amsterdam 1117, The Netherlands; Amsterdam Public Health, Mental Health Program, Amsterdam, The Netherlands; Department of Psychiatry, Amsterdam UMC Location Vrije Universiteit Amsterdam, Boelelaan, Amsterdam 1117, The Netherlands; Amsterdam Public Health, Mental Health Program, Amsterdam, The Netherlands; Amsterdam Public Health, Methodology Program, Amsterdam, The Netherlands; Amsterdam Neuroscience, Mood, Anxiety, Psychosis, Sleep & Stress Program, Amsterdam, The Netherlands; Amsterdam Neuroscience, Complex Trait Genetics, Amsterdam, The Netherlands; Department of Psychiatry, Amsterdam UMC Location Vrije Universiteit Amsterdam, Boelelaan, Amsterdam 1117, The Netherlands; Amsterdam Public Health, Mental Health Program, Amsterdam, The Netherlands; Department of Clinical Psychology, University of Amsterdam, Amsterdam, The Netherlands; Department of medical Psychology, Amsterdam University Medical Centers, Amsterdam, The Netherlands; Department of Psychiatry, Amsterdam UMC Location Vrije Universiteit Amsterdam, Boelelaan, Amsterdam 1117, The Netherlands; Amsterdam Public Health, Mental Health Program, Amsterdam, The Netherlands; Amsterdam Public Health, Methodology Program, Amsterdam, The Netherlands; Central Diagnostic Laboratory, Division Laboratories, Pharmacy, and Biomedical Genetics, University Medical Center Utrecht, Utrecht University, The Netherlands; Department of Genomic Sciences, University of Virginia, Charlottesville, VA, USA; Department of Clinical Chemistry, Faculty of Medicine and Health Technology, Tampere University, Tampere, Finland; Faculty of Medicine and Health Technology, Finnish Cardiovascular Research Center Tampere, Tampere University, Tampere, Finland; Department of Clinical Chemistry, Fimlab Laboratories, Tampere, Finland; Department of Clinical Chemistry, Faculty of Medicine and Health Technology, Tampere University, Tampere, Finland; Faculty of Medicine and Health Technology, Finnish Cardiovascular Research Center Tampere, Tampere University, Tampere, Finland; Department of Clinical Chemistry, Fimlab Laboratories, Tampere, Finland; Faculty of Medicine and Health Technology, Finnish Cardiovascular Research Center Tampere, Tampere University, Tampere, Finland; Department of Clinical Physiology, Tampere University Hospital, Tampere, Finland; Research Centre of Applied and Preventive Cardiovascular Medicine, University of Turku, Turku, Finland; Department of Clinical Physiology and Nuclear Medicine, Turku University Hospital, Turku, Finland; Centre for Population Health Research, University of Turku and Turku University Hospital, Turku, Finland; Unit of Medical Technology and Intelligent Information Systems, Department of Materials Science and Engineering, University of Ioannina, Ioannina, Greece; Biomedical Research Institute, Foundation for Research and Technology - Hellas (FORTH), Ioannina, Greece; Computational Science Lab, Institute of Informatics, University of Amsterdam, Amsterdam, The Netherlands

**Keywords:** Comorbidity, Depression, Cardiovascular diseases, Network analysis

## Abstract

**Aims:**

Prevalence of cardiovascular diseases (CVDs) and depression is rising globally. Their co-occurrence associates with poorer outcomes, potentially due to shared metabolic pathways. This study aimed to identify metabolic pathways linking depression symptoms and CVD risk factors.

**Methods and results:**

Data from the Young Finns Study (YFS, *n* = 1,599, mean age 37 ± 5, 54% female) served as input for a network (mixed graphical models). Confirmatory analysis through covariate-adjusted regression was done with UK Biobank (UKB, *n* = 69,513, mean age 63 ± 7, 64% female). Mendelian randomization assessed causality using genome-wide association studies data. The study examined 52 plasma metabolites measured by nuclear magnetic resonance spectroscopy. Outcomes included depression symptoms (BDI in YFS, PHQ-9 in UKB) and CVD risk factors [systolic/diastolic blood pressure, carotid intima–media thickness (cIMT)]. Mendelian randomization inferred causal links between metabolites and depression or (intermediate markers of) CVD. Two bridge metabolites were identified: glucose linked to sleep pattern (*P* = 0.034); omega-3 fatty acids (FAs) linked to appetite change (*P* < 0.001); and both connected to cIMT (both *P* = 0.002). Mendelian randomization suggested glucose as causal in coronary artery disease (CAD) risk, while omega-3 FAs showed potential causal links to CAD, coronary artery calcification, and cIMT.

**Conclusion:**

This study integrated three statistical techniques and identified two metabolic markers (glucose, omega-3 FAs) connecting depression and CVD on a symptom and risk factor level. The associations, established in a relatively young cohort, were replicated in a predominantly middle-aged cohort and emphasize both the generalizability of the findings across different populations and value of symptom-level analysis in depression and CVD comorbidity research.

## Structured Graphical Abstract

**Structured Graphical Abstract:**
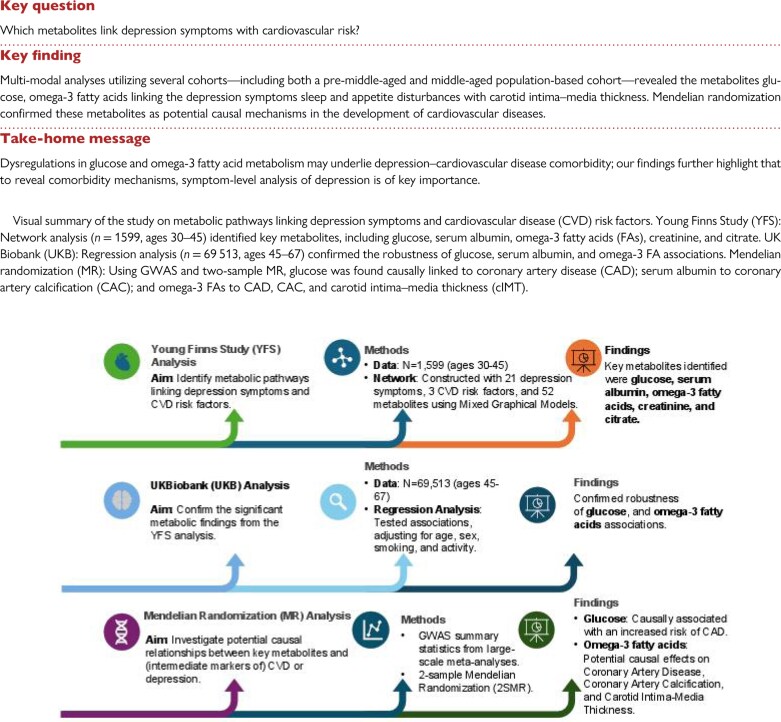

## Introduction

Cardiovascular diseases (CVDs) and major depressive disorder (MDD) frequently co-occur, and these conditions share a high burden of disease, are often chronic, and both show a steady rise in incidence globally.^1^ Advancements in metabolomics have provided substantial progress in elucidating the biology of both diseases.^2–4^ Nuclear magnetic resonance (NMR)–based metabolomics has related depression to shifts in lipids, fatty acids (FAs), and low-molecular-weight metabolites.^3^ Individuals with MDD exhibit disturbed energy metabolism, including reduced citrate and elevated pyruvate level.^5^ Furthermore, evidence suggests a potential causal link between omega-3 FAs and MDD, alongside broader metabolic alterations.^6^ These metabolites may thus provide intervention targets to curtail the onset, severity, and progression of depression.^7,8^ Similarly, metabolomic studies of CVD identified unique signatures linked to CVD.^9–11^ These include associations between lipoproteins, especially very LDL (VLDL), cholesterol, triglycerides, and glycoprotein acetyls, with increased CVD risk.^12^

When depression and CVD co-occur, the medical prognosis of each condition worsens, marked by higher disease severity and increased mortality risk.^13,14^ Metabolomic analyses may likewise aid in understanding their substantial comorbidity.^15^ However, this approach remains underexplored as research has predominantly delved into each ailment in isolation. This fragmented approach has limited our understanding of possible shared metabolic pathways and aetiology.^16–18^ An integrative approach is needed to explore the interconnected metabolic processes underlying both conditions.

In this study, we present a multi-modal data analysis framework for comorbidity research, providing tools to link diverse types of data to distinct phenotypes. Using this approach, we aimed to identify metabolites linking both morbidities through network analysis. Network analysis enabled a comprehensive overview of all potential associations. Utilizing depression symptoms as the unit of analysis rather than relying on a binary clinical diagnosis enabled us to better capture the heterogeneous nature of depression.^19^ It is important to note that these analyses are exploratory, and inferences cannot be made regarding depressive symptoms and metabolites.

For the analyses, we utilized population data on pre-middle-aged adults (aged 30–45), detecting comorbidity risk markers (rather than CV incident) that manifest already in early life. These findings were then validated using a data set of middle-aged adults (aged 45–67), ensuring the robustness and generalizability of identified comorbidity markers across the life course. To infer potential causality, identified markers were subsequently included in a Mendelian randomization (MR) analysis, using genetic variants as instrumental variables to minimize confounding bias.^20^

## Methods

### Data Sets

In the present study, we made use of three different data sets. The Young Finns study (YFS) served as input for the network analysis^21^; the UK Biobank (UKB)^22^ data were used for robustness testing. The UKB provides a robust data set for external validation of YFS findings, featuring key variables like metabolic biomarkers quantified using the same NMR platform as YFS. We used several sources for summary statistics from genome-wide association study (GWAS) data for the MR procedure.

### The Young Finns Study

The YFS is a population-based prospective cohort study carried out at five medical schools in Finland (Turku, Helsinki, Kuopio, Tampere, and Oulu). The YFS aimed to thoroughly evaluate cardiovascular risk factors in children and adolescents across the nation. In the 2007 follow-up, the study included data from approximately 2200 individuals aged 30–45 years, who had been followed since childhood. We considered those participants whose NMR-measured metabolic data, depression, and CVD risk factors assessment were available in this 2007 wave (*n* = 1599). For more extensive information on subject selection, we refer to the Supplementary material. In total, analyses included 21 depression symptoms, 3 CVD risk factors (referred to as phenotypes), and 52 metabolites to create a network with 76 variables (nodes).

### Metabolites

Using serum samples, 229 metabolic parameters were quantified using a high-throughput NMR metabolomics platform (Nightingale Health, Helsinki, Finland).^23^ For metabolic categorization, we refer to the Supplementary  material online, *Materials* and *Table S1*. This resulted in a selection of 52 metabolites and was drawn from literature on metabolites in depression and CVD risk factors.^4,24^

### Depression symptoms

Depression symptoms were assessed using a revised version of the Beck Depression Inventory (BDI-II).^25^ The BDI-II contains 21 questions measuring characteristic symptoms of depression experienced over the past 2 weeks (see Supplementary material online, *Materials* and *Table S2* for details). The analysis included each of these items individually, rather than using a summary score, to provide a more detailed assessment of the participants’ depression symptomatology beyond the overall BDI score.^26^

### Cardiovascular disease risk factors

We selected a set of three CVD risk factors (see Supplementary material online, *Table S3* for details), aiming to cover a broad spectrum of potential early risk markers of cardiovascular health. These include systolic and diastolic blood pressure (SBP, DBP, respectively) and an attribute displaying average carotid intima–media thickness (cIMT). Carotid intima–media thickness measures the thickness of the inner two layers of the carotid artery, the intima and media. The inclusion of cIMT as a cardiovascular risk factor is justified by its ability to detect early atherosclerotic changes, its strong predictive value for cardiovascular events.^27–29^

### Covariates

Models were adjusted for differences in sex and age before constructing the network. We did this by first applying an ANOVA test between the network nodes (variables) and sex and age separately. Variables with significant associations were then adjusted for the relevant covariate(s).

### UK Biobank

To check the robustness of the significant metabolites associated with depression symptoms and CVD risk factors identified in the YFS, we used data from the UKB. The sample consisted of 157 286 participants and contained information on depression symptoms (Patient Health Questionnaire-9 or PHQ-9^30^), CVD risk factors, and the same metabolic platform used in the YFS (see Supplementary material online, *Tables S4* and *S5*). Covariates included sex, age, smoking status, and physical activity (see Supplementary material online, *Materials* for extensive information).

### Genome-wide association study summary statistics for Mendelian randomization

For each metabolite and CVD phenotype, summary statistics were obtained from large GWAS meta-analyses (listed in Supplementary material online, *Table S6*). Disease phenotypes included coronary artery disease (CAD), ischaemic stroke subtypes. No GWAS data were available for individual depression symptoms; therefore, GWAS data for MDD were selected. These GWAS summary statistics served as input data for the MR analysis.

### Pre-processing analysis

The pre-processing analysis involved data imputation. To address the missing phenotype data, multiple imputation (MI) was used using iterative imputer from sklearn in python version 3.11.4. using 10-fold imputation with random forests.^31,32^ This algorithm capitalizes on the interrelationships among features to provide more precise estimates for missing values and is known to handle outliers and skewness well. To evaluate the effectiveness of the imputation, we calculated descriptive statistics for each column in both the complete and imputed data sets.

### Main analysis

Analyses were conducted in R (version 4.1.3). The ‘mgm’ (version 1.2–12)^33^ and ‘qgraph’ (version 1.9.4)^34^ packages for the R statistical software were used. The network visualizations in this study were generated using the Gephi software (version 0.10),^35^ a powerful open-source tool for network analysis and visualization.

### Mixed graphical models

Depression symptoms (ordinal) along with CVD risk factors (which could be categorical or continuous) and metabolite levels following a Gaussian distribution (see Supplementary material online, *Figure S1* for metabolite distributions) were inputted into the network model using the mgm package, after checking for sex/age dependence using an ANOVA test, resulting in a network with 76 nodes (see Supplementary material online, *Methods* for more details).

### Stability analysis

We conducted a stability analysis using bootstrapping, resulting in a measure for edge stability with a 95% confidence interval and a fraction of times an edge appeared in the bootstraps. For further clarification, we refer to the Supplementary material.^4,36^ To further assess the robustness of associations between depressive symptoms, CVD risk factors, and metabolites, we conducted a permutation test with 1000 iterations (for detailed methodology, see Supplementary material).^37^

### Centrality and jointness (comorbidity) assessment

In the analysis, node importance within the sub-network of metabolites was assessed using two metrics: degree centrality and the jointness score. These two measures together show the importance of a node in the network system and give an indication of which metabolites might have the highest influence in connecting depression symptoms with CVD risk factors. More explanation for the definition and rationale of these measures can be found in the Supplementary material online, *Methods*.

We aimed to select metabolites scoring low for degree centrality and high for jointness score. The rationale behind this is that metabolites with high degree centrality may be involved in numerous biological pathways and associated with multiple diseases (pleiotropy), and by selecting lower degree centrality metabolites, we avoid confounding due to involvement in other conditions. Having high jointness score implies a metabolite might be specifically relevant to both CVD risk factors and depression symptoms, which can provide more targeted insights into the shared metabolic pathways. By identifying metabolites that are uniquely significant to both depression symptoms and CVD risk factors, we can improve research on screening, diagnostics, and intervention, thereby enhancing precision medicine.

### Robustness analysis

After assessing stability and centrality, we checked whether associations disappeared after correcting for covariates. To achieve this, an ordinary least squares (OLS) model was applied in an external data set (UKB) for each variable connected to the metabolites filtered out by the stability and centrality analysis. We corrected for sex, age, smoking status, and physical activity in various combinations, which were not accounted for in the network analysis.

### Mendelian randomization

As an additional analysis step, we selected the stable, central, and robust metabolites from the network and performed two-sample MR (2SMR) based on GWAS summary statistics.^38–42^ We tested the potential causal relationship between metabolites and (intermediate markers of) CVD and bidirectional causal relationships between metabolites and depression; MR is a powerful statistical tool allowing to infer potential causal links (for further explanation of and elaboration on the methodological concepts, we refer to the Supplementary material). The phenotypes and metabolites used in the analysis can be found Supplementary material online, *Table S6*^43–47^ along with their sample sizes. For more detailed methodology, refer to the Supplementary material online, *Methods*. All F statistics > 10 indicated that the strength of selected genetic instruments was adequate (see Supplementary material online, *Table S7*).^48^ Sensitivity analyses were based on weighted median and MR-Egger estimators. Cochran’s *Q* test was conducted to identify heterogeneity among SNPs and the MR-Egger intercept examined for pleiotropic effects.

## Results

### Pre-processing

The YFS sample consisted of 1599 subjects (54% female); 4.6% had a BDI score of >19 and 1.7% used statins; for further details, we refer to *Table 1*. Details on the BDI items are available in *Table 2* and CVD risk factor items in Supplementary material online, *Table S3*; missingness is provided in Supplementary material online, *Table S8*; depression symptoms exhibited around 21% missing data, while CVD risk factors had minimal missingness (below 1%). Information on imputation results is in Supplementary material online, *Figure S2*. The ANOVA results for the associations between variables and sex/age are shown in Supplementary material online, *Figure S3*. These data were fed to the mixed graphical model (MGM).

**Table 1:** Descriptive statistics for the 2007 Young Finns Study nuclear magnetic resonance subset

Characteristics	*n* = 1599 (YFS subset with complete NMR and BDI data)
Socio-demographic	
** **Sex (F) (%)	54
Age, years (mean ± SD)	37.8 ± 5
Education level (%)	
** **Low	4.7
** **Intermediate	70.5
** **High	24.7
Health indicators	
** **BMI (mean ± SD)	26.2 ± 6
** **Waist circumference, cm (mean ± SD)	89.1 ± 13
** **Hip circumference, cm (mean ± SD)	100 ± 8.8
** **Waist-to-hip ratio (mean ± SD)	0.9 ± 0.08
** **CVD history (%)	30
** **Hypertension (%)	6.3
** **Diabetes (%)	1.2
Metabolic and lifestyle factors	
** **Metabolic syndrome (%)	18.14
** **Total cholesterol, mmol/L (mean ± SD)	5.1 ± 0.89
** **LDL cholesterol, mmol/L (mean ± SD)	3.2 ± 0.78
** **HDL cholesterol, mmol/L (mean ± SD)	1.3 ± 0.3
** **Triglycerides, mmol/L (mean ± SD)	1.4 ± 0.76
** **Remnant cholesterol (non-HDL07, non-LDL cholesterol) (mean ± SD)	1.7 ± 0.44
** **Smokers (%)	18.9
** **Alcohol intake per day (mean ± SD)	0.88 ± 1.2
** **Exercise (mean ± SD)	19.2 ± 21.45
Depression symptoms	
** **BDI score > 19 (%)	4.6
Medication use	
** **Antidepressant (%)	6.3
** **Statins (%)	1.7

**Table 2:** Regression analysis of metabolites was performed with adjustment for multiple covariate sets *P*-values from external validation in the UK Biobank cohort are shown, with statistically significant associations (*P* < 0.05) indicated in bold

Dependent variable	Independent variable	Covariate(s)	Beta	*P*-value
Serum albumin	Trouble sleeping	–	**−0.0088**	**0**.**0053**
		Age	**−0**.**0120**	**<0**.**001**
		Gender	−0.0040	0.1100
		Age, gender	**−0**.**0083**	**<0**.**005**
		Age, gender, smoking status	**−0**.**0070**	**<0**.**005**
		Age, gender, smoking status, physical activity	**−0**.**0073**	**0**.**0083**
	Intima media thickness	–	−0.0018	**<0**.**005**
		Age	−0.0027	**<0**.**005**
		Gender	−0.0017	**0**.**0051**
		Age, gender	−0.0026	**<0**.**005**
		Age, gender, smoking status	−0.0026	**<0**.**005**
		Age, gender, smoking status, physical activity	−0.0026	**<0**.**005**
Glucose	Insomnia	–	**0**.**0200**	**<0**.**001**
		Age	**0**.**0120**	**<0**.**001**
		Gender	**0**.**0170**	**<0**.**001**
		Age, gender	**0**.**0083**	**0**.**0180**
		Age, gender, smoking status	**0**.**0080**	**0**.**0100**
		Age, gender, smoking status, physical activity	**0**.**0082**	**0**.**0200**
	Trouble falling or staying asleep or sleeping too much	–	**−0**.**0002**	**0**.**0100**
		Age	−0.0001	0.0640
		Gender	**−0**.**0002**	**<0**.**01**
		Age, gender	−0.0001	0.0630
		Age, gender, smoking status	−0.0001	0.0600
		Age, gender, smoking status, physical activity	−0.0001	0.0640
	Intima media thickness	–	**0**.**8500**	**<0**.**001**
		Age	**0**.**5300**	**0**.**0160**
		Gender	**0**.**8600**	**<0**.**001**
		Age, gender	**0**.**5400**	**0**.**0150**
		Age, gender, smoking status	**0**.**5400**	**0**.**0150**
		Age, gender, smoking status, physical activity	**0**.**5300**	**0**.**0160**
Creatinine	Recent lack of interest or pleasure in doing things	–	**0**.**0001**	**0**.**0140**
		Age	**0**.**0002**	**<0**.**005**
		Gender	0.0001	0.2100
		Age, gender	0.0001	0.1100
		Age, gender, smoking status	0.0001	0.1000
		Age, gender, smoking status, physical activity	0.0001	0.1100
	Insomnia	–	**−0**.**0370**	**<0**.**001**
		Age	**−0**.**0450**	**<0**.**001**
		Gender	**0**.**0082**	**0**.**0140**
		Age, gender	0.0026	0.4300
		Age, gender, smoking status	0.0030	0.3600
		Age, gender, smoking status, physical activity	0.0068	0.4300
	Sleeping change	–	**−0**.**069**	**<0**.**0001**
		Age	**−0**.**057**	**<0**.**0001**
		Gender	−0.0013	0.8800
		Age, gender	0.0062	0.4700
		Age, gender, smoking status	0.0060	0.4000
		Age, gender, smoking status, physical activity	0.0027	0.4300
	Intima media thickness	–	0.0030	0.2400
		Age	0.0020	0.4400
		Gender	0.0030	0.2500
		Age, gender	0.0020	0.4400
		Age, gender, smoking status	0.0020	0.4400
		Age, gender, smoking status, physical activity	0.0020	0.4200
Omega-3 fatty acids	Recent poor appetite or overeating	–	<0.0001	0.6800
		Age	0.0001	0.2700
		Gender	0.0001	0.5200
		Age, gender	0.0001	0.1600
		Age, gender, smoking status	0.0001	0.1500
		Age, gender, smoking status, physical activity	0.0001	0.1600
	Intima media thickness	–	**0**.**1730**	**<0**.**001**
		Age	0.0940	0.0690
		Gender	**0**.**1740**	**<0**.**001**
		Age, gender	0.0950	0.0670
		Age, gender, smoking status	0.0940	0.0670
		Age, gender, smoking status, physical activity	0.0950	0.0650
Citrate	Trouble sleeping	–	−0.0031	0.2500
		Age	0.0023	0.4000
		Gender	**−0**.**0110**	**<0**.**001**
		Age, gender	<−0.0001	0.7800
		Age, gender, smoking status	−0	0.8200
		Age, gender, smoking status, physical activity	0	0.8200
	Diastolic blood pressure	–	**0**.**0002**	**<0**.**001**
		Age	**0**.**0002**	**<0**.**001**
		Gender	0.0002	**<0**.**001**
		Age, gender	0.0002	**<0**.**001**
		Age, gender, smoking status	0.0002	**<0**.**001**
		Age, gender, smoking status, physical activity	0.0002	**<0**.**001**

### Network description

**Figure 1:**
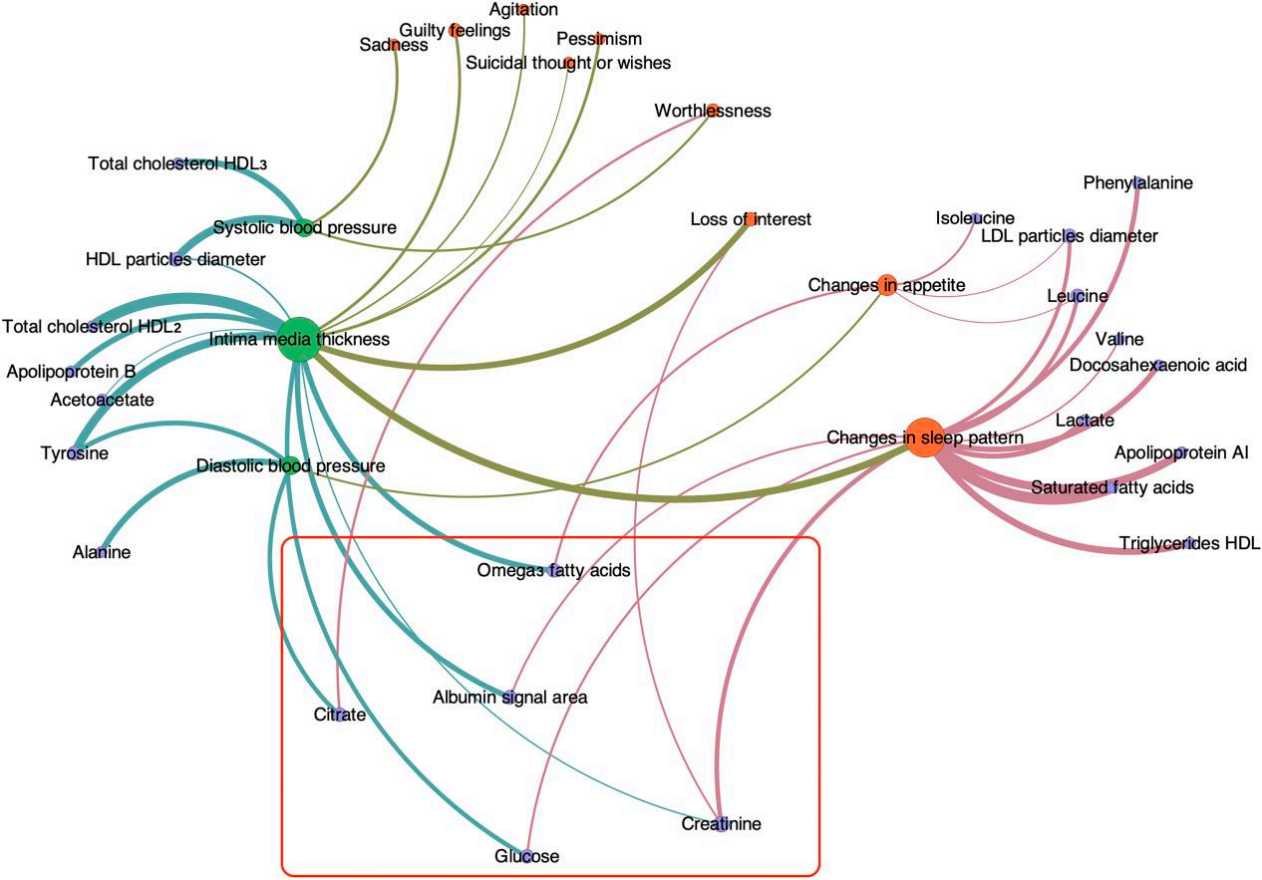
Network visualization displaying only the connections between depression symptoms, selected metabolites, and cardiovascular disease risk factors, with intra-group connections omitted for clarity. Metabolites shared between depression symptoms and intra-group risk factors are highlighted within the red box.

The MGM created a network (*Figure 1*) with 76 nodes consisting of 21 BDI items (orange), 52 metabolites (purple), and 3 CVD risk factors (green). The inter-group connections were stronger than the intra-group connections and were omitted in the visualization of the network. The metabolites serving as a bridge between CVD risk factors and BDI items were omega-3 FAs, creatinine, albumin signal area, glucose, and citrate. In total, there were six metabolic pathways (five metabolites in total): (i) cIMT, omega-3 FAs, and change in appetite; (ii) cIMT, creatinine, and loss of interest; (iii) cIMT, creatinine, and change in sleep pattern; (iv) cIMT, albumin, and change in sleep pattern; (v) cIMT, glucose, and change in sleep pattern; and (vi) DBP, citrate, and worthlessness.

### Network and robustness analysis

**Figure 2:**
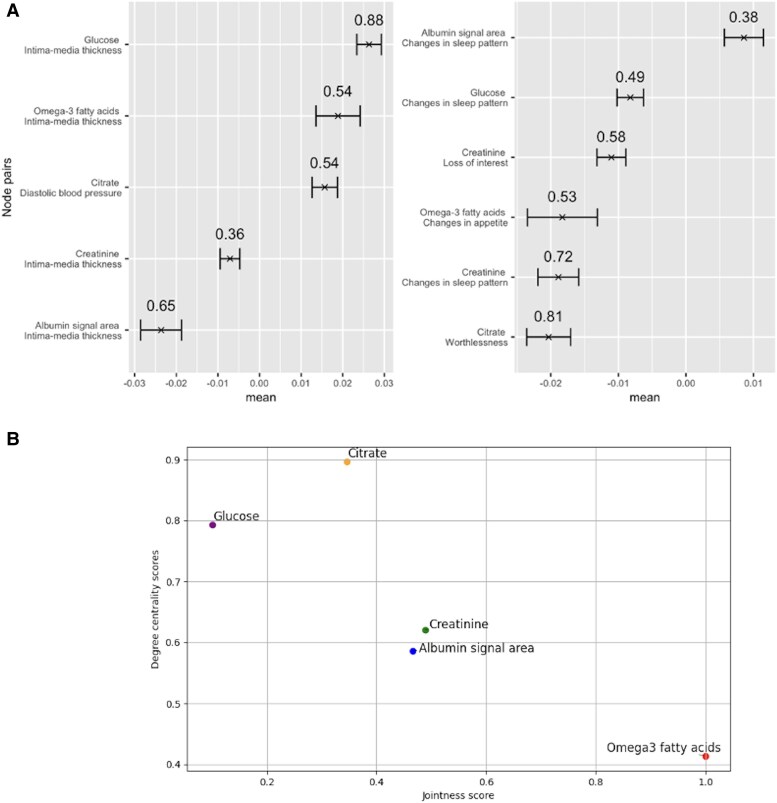
(*A*) Stability analysis of the metabolite–symptom and metabolite–risk factor pairs, showing the pairs where metabolites are linked to both a symptom and a risk factor. The *x*-axis shows the average edge weight of the node pairs, computed by bootstrapping the network 100 times, with the minimum and maximum edge weight presented as an interval. The pairs are ranked by edge weight, with the highest weights at the top. The fraction of times the edge was present across bootstrapped samples is displayed above the intervals. Higher values imply more stability for perturbations in the system. (*B*) Scatter plot of the stable metabolites, with the *x*-axis representing the jointness score and the *y*-axis representing degree centrality, highlighting the relationship between these two metrics. A higher jointness score indicates more influence on the system, whereas a lower degree centrality implies higher susceptibility for changes in the system; in this figure, omega-3 fatty acids are most ‘influential’ on the system according to our metric, followed by albumin/creatinine, and then glucose and citrate.

The 6 pathways corresponded to 12 metabolite–phenotype pairs (the network’s ‘edges’); we tested the stability of these edges (*Figure 2A*) and assessed the metabolites’ degree centrality vs. jointness score (*Figure 2B*). Albumin was slightly unstable, but relatively central; glucose was more stable, but not central. Omega-3 FAs were semi-stable and highly central. Creatinine was slightly unstable and semi-central; and citrate was slightly unstable and not central.

**Figure 3:**
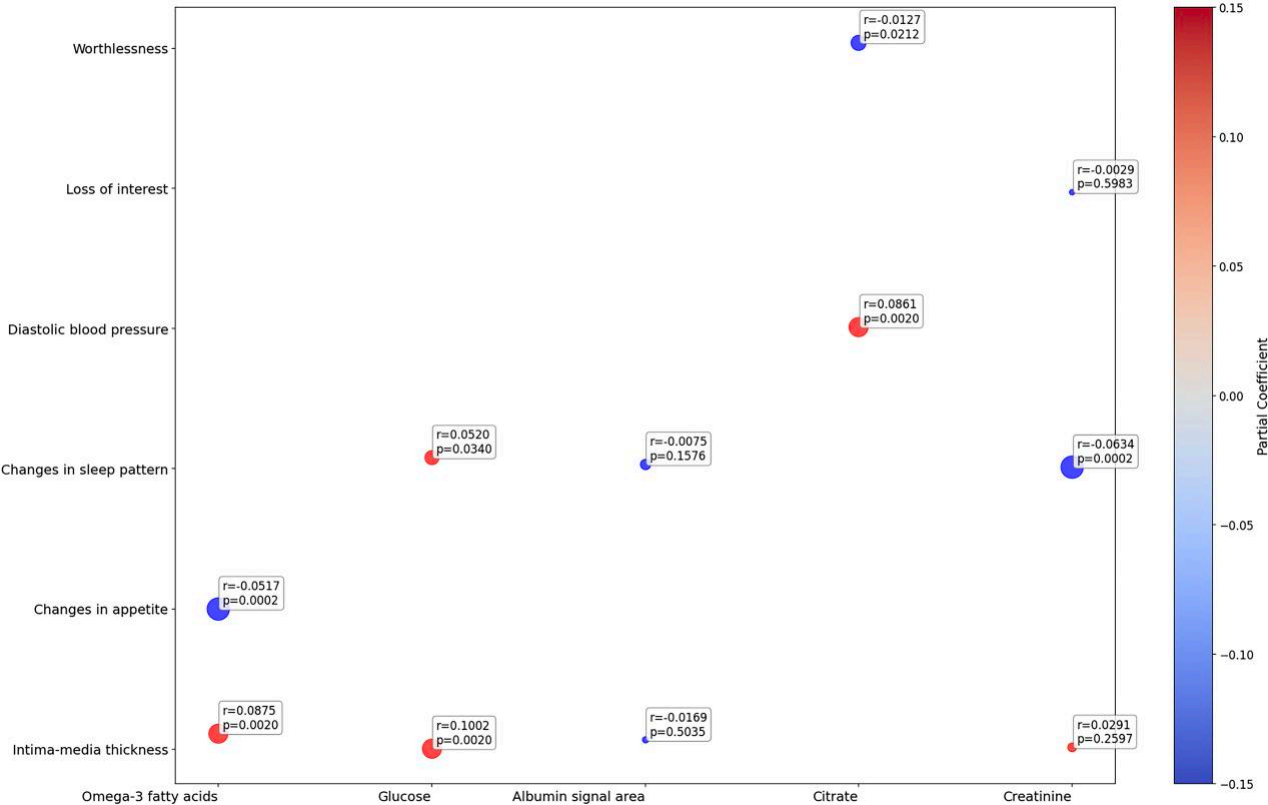
Plot illustrating the shared metabolites identified between depressive symptoms and cardiovascular disease risk factors after permutation testing (1000 iterations). The *y*-axis represents depressive symptoms and cardiovascular disease risk factors, while the *x*-axis displays the shared metabolites. Each point corresponds to a symptom–metabolite or cardiovascular disease risk–metabolite pair, with the *r*-value (partial correlation) and *P*-value shown for each association.

After permutation testing, we identified three robust bridge metabolites linking depression and CVD risk (*P* < 0.05): omega-3 FAs, glucose, and citrate. Omega-3 FAs showed significant connections to IMT (*r* = 0.0875, *P* = 0.002) and changes in appetite (*r* = −0.0517, *P* < 0.001). Glucose also demonstrated associations with IMT (*r* = 0.1002, *P* = 0.002) and changes in sleep pattern (*r* = 0.052, *P* = 0.034). Citrate was found to be connected to DBP (r = 0.0861, *P* = 0.002) and feelings of worthlessness (*r* = 0.0127, *P* = 0.0212) (*Figure 3*).

Additionally, we tested all metabolite–phenotype pairs for robustness using OLS in UKB (*Table 2*). We regressed, correcting for nothing; age; sex; age and sex; age, sex, and smoking status; and age, sex, smoking status, and physical activity. Creatinine was not robust; citrate was not robust with feeling worthless but was with DBP. Omega-3 FAs were not robust with change in appetite which might be due to countering effects of increased vs. decreased appetite; the connection with cIMT was not robust after correcting for age. We found glucose to be consistently robust with their phenotypes. Based on the stability analysis, centrality assessment, and robustness analysis, we selected metabolites that scored at least semi-high on two out of the three assessments (see Supplementary material online, *Table S9*). This resulted in selecting glucose and omega-3 FAs for MR.

### Mendelian randomization

After performing MR on the selected metabolites, we identified several potential causal relationships between metabolites and both cardiometabolic traits and depression phenotypes. The results indicated that genetically predicted higher levels of glucose were associated with increased risk CAD (OR = 1.14 ± 0.10, *P* = 0.00421). The same procedures and analyses were performed on the depression and intermediate markers of CVD; using omega-3 FAs as exposure, results indicated a significant association between the metabolite and increased risk of CAD (OR = 1.33 ± 1.60, *P* = 7.04 × 10^−15^), CAC (OR = 1.50–1.95, *P* = 1.24 × 10^−15^), and cIMT (OR = 1–1.02, *P* = 0.0303). In direction and effect size, estimates obtained using the weighted median and the MR Egger method were consistent with the significant IVW estimates across metabolites (see Supplementary material online, *Table S10*). Cochran’s *Q* indicated evidence of heterogeneity between SNPs for the effect of glucose on CAD and omega-3 FAs on CAD, cIMT, and CAC (see Supplementary material online, *Table S11*). However, pleiotropy did not appear to be an issue (see Supplementary material online, *Table S12*). There was no evidence for a potential causal effect of genetically predicted levels of the three selected metabolites on depression. In reversed MR analyses, where depression was considered the exposure and the outcomes were glucose and omega-3 FAs levels, the MR results did not indicate any potential causal effect of depression genetic liability on these metabolites (see Supplementary material online, *Table S10*).

## Discussion

Applying a multi-modal data analysis framework, this study aimed to identify metabolic markers linking depression symptoms and CVD risk. The key innovation lies in the use of network analysis to connect CVD risk factors and depression symptoms, which are typically studied separately. By integrating network analysis with OLS-covariate adjustment and MR, we further enhanced the reliability of our findings, distinguishing potential causal links. Glucose, serum albumin, omega-3 FAs, creatinine, and citrate served as bridges. Glucose linked cIMT with changes in sleep patterns; omega-3 FAs linked cIMT and changes in appetite. The OLS analyses indicated robustness over the life course and over depression assessment instruments. While UKB data on incident cardiovascular events could provide valuable insights into long-term outcomes, our study focused on early metabolic markers linking depression and CVD, leveraging the younger YFS cohort (mean age 37) to explore early-stage mechanisms of comorbidity. Subsequent causal inference (MR) analyses indicated causal relationships between the metabolites and CVD outcomes (namely, CAD) and intermediate CVD outcomes (namely, CAC and cIMT).

Glucose, a primary energy source, circulates in blood to supply tissues. Dysregulation of glucose metabolism characterizes diabetes mellitus, leading to high fasting glucose and potential tissue damage. This dysregulation is linked to various organ diseases^49,50^ and mental health conditions like depression.^51–55^ Our findings showed that glucose levels was linked to increased comorbidity. Thus, glucose regulation is crucial for both physical and mental health. Recent metabolomics studies have also highlighted the role of glucose dysregulation in both depression and cardiovascular health. For instance, recent study found that elevated inflammatory markers and metabolic dysregulation might mediate the bidirectional relationship between depression and CVD.^56^ Our findings on glucose as a key metabolite linking cIMT to changes in sleep patterns provide further evidence for this connection.

Omega-3 FAs served as key metabolite. Omega-3 FAs are polyunsaturated FAs involved in animal lipid metabolism. They are a vital part of cell membranes and provide structure and support in interactions between cells.^57^ They are associated with health benefits such as reduced inflammation, triglyceride levels, and blood pressure^58,59^ and even reduction of depression symptoms,^60^ although evidence shows heterogeneous results.^61,62^ Evidence of omega-3 FA dietary supplementation supporting cardiovascular/mental health benefits is equivocal.^63–66^ While some studies have reported significant benefits of omega-3 FAs on reducing inflammation and depressive symptoms,^67^ others have found more heterogeneous results depending on dosage, formulation, or patient characteristics.^60^ These disparate findings are consistent with our observation of medium stability for omega-3 FAs in centrality assessments and evidence of pleiotropy in MR analyses.

Our results align with the hypothesis of immunometabolic depression, which states that low-grade inflammation and metabolic dysregulations associate with energy-related symptoms of depression.^4,19^ In turn, these metabolites may be involved with development of CVD.^68–70^ For example, a study by de Kluiver *et al*.^71^ identified metabolomic profiles associated with atypical depression symptoms, including fatigue, hypersomnia, and increased appetite—symptoms that overlap with those linked to glucose dysregulation in our study. Similarly, Rydin *et al*.^4^ used network analysis to explore connections between depressive symptoms and metabolites, identifying fatigue and hypersomnia as central nodes. While their study focused more on symptom-level associations, our work extends these findings by identifying specific metabolites—such as glucose and omega-3 FAs—as bridges between depression symptoms and CVD risk factors. Specifically, we found links between depression symptoms related to energy bridged by several metabolites to mainly cIMT. We could however not distinguish increases in appetite and sleep from decreases as only changes in appetite and sleep were available in the data. Increased but not decreased appetite and sleep have been hypothesized to be part of immunometabolic depression concept.^72^

A few limitations are warranted: the network connections between several metabolites and changes in appetite and sleep symptoms were relatively weak. This may be because an association may differ for directions of symptom change—such as increased vs. decreased appetite or sleep. For example, increased appetite, but not decreased appetite, is linked to poorer metabolic health and differing processes for increased sleep vs. insomnia in depressed individuals have also been observed.^4,19,73^ Another limitation was availability of GWAS data being on only depression, whereas the nature of this current work highlighted individual depression symptoms may be differentially linked to certain metabolic alterations. Cross-sectional nature of the primary analysis limits causal inferences. Mendelian randomization analyses suggested potential causal connections between metabolites and certain outcomes. Nevertheless, since the phenotype used in MR analyses was different as compared to those used in main analyses and certain MR results indicated the presence of horizontal pleiotropy, the causality in the associations that emerged in the main analyses remains to be proven.

For future work, we recommend expanding network analyses to include the full spectrum of metabolites available from the NMR platform, rather than limiting the study to the current subset, or to utilize network methods that allow for nonlinear relationships. Additional to methodological recommendations, we suggest exploring the mechanistic role of glucose and omega-3 FAs, as to deepen the understanding how cardiovascular health (with a specific focus on cIMT, CAD, and CAC) can be screened and improved.

The identification of glucose and omega-3 FAs as metabolites associated with depression–CVD comorbidity (specifically, cIMT, CAD, and CAC) highlighted the shared metabolic pathways and the potential of metabolomic profiling in clinical care. These findings align with the previous research linking glucose dysregulation to both CVD and depression.^53,74–76^ Furthermore, these results confirm the varying effects of omega-3 FAs on cardiovascular and mental health.^77,78^ The relationship between glucose/omega-3 FAs and CVD risk factors is not linear or straightforward; rather, it is complex and context-dependent. This complexity underscores the need for further investigation into subgroups that exhibit similar patterns of glucose, omega-3 FAs, depression, and CVD. Such analysis may inform more targeted treatment strategies. Specifically, some individuals may present with lower glucose levels but higher depression, which could indicate a need for interventions for CAD, despite the lower glucose level, which is typically considered favourable when within normal ranges.

## Supplementary Material

oeaf038_Supplementary_Data

## Data Availability

In accordance with the data usage agreements of the YFS and UKB, the data sets supporting the conclusions of this article are not available for public access. Both YFS and UKB impose strict conditions on the confidentiality and use of their data, which prohibit the sharing of individual-level data. The data from these sources have been utilized under specific conditions that ensure privacy and adherence to ethical guidelines. Consequently, access to the raw data used in this study is restricted to the research team, as approved by the respective data custodians. Researchers interested in accessing data from YFS or UK Biobank are encouraged to apply directly to the respective organizations. The summary statistics obtained from large GWAS meta-analyses (listed in Supplementary material online, *Table S6*) are publicly available.

## Lead author biography

Angela Koloi is a data scientist pursuing her PhD at the intersection of computational science and clinical medicine. Her PhD research at the University of Amsterdam and University of Ioannina develops novel causal inference and machine learning approaches to unravel the immunometabolic underpinnings of cardiovascular–depression comorbidity. She employs an innovative methodological spectrum—from network analysis to AI techniques—applied to large-scale cohorts to decode the biological dialogue between metabolic health and mental well-being. With dual training in cardiovascular biomechanics (MSc: stent-atherosclerosis simulations) and industry experience in causal AI, she bridges engineering precision with clinical research. Her work, supported by EU Horizon 2020 TO_AITION, exemplifies how multidisciplinary approaches can unravel complex disease comorbidities.

Arja Rydin has an interdisciplinary background, having studied liberal arts and sciences and majoring in mathematics. The focus on interdisciplinary research carried on to applying computational science on mental health–related questions in her master and resulted in pursuing a PhD studying the comorbidity of depression and cardiovascular diseases developing and applying novel analytical approaches. These include the combination of classical statistical approaches such as regression analyses, but also machine learning and network analyses, covering both cross-sectional and longitudinal data sets. She believes intersecting different disciplines can greatly aide in unravelling mechanisms of the ‘body and mind’ connection.
