# Supplementary material for: Morbidity-bridging metabolic pathways: linking early cardiovascular disease risk and depression symptoms using a multi-modal approach
Source: Eur Heart J Open. 2025 Apr 16;5(3):oeaf038. doi: 10.1093/ehjopen/oeaf038 (PMC12053008; doi:10.1093/ehjopen/oeaf038)
Supplement: oeaf038_Supplementary_Data [file oeaf038_supplementary_data.docx]

**Supplemental Online Content**

Morbidity-bridging metabolic pathways: linking early cardiovascular disease risk and depression symptoms using a multi-modal approach

**Authors:** Angela Koloi 1,2,3*, Arja Rydin4,5*, Yuri Milaneschi4,5, 6, 7,8, Femke Lamers4,5, Jos A. Bosch3,9, Emma Pruin4,5,6, Sander W. van der Laan10,11, Pashupati P. Mishra12,13,14, Terho Lehtimäki12,13,14, Mika Kähönen13,15, Olli T. Raitakari16,17,18, Dimitrios I. Fotiadis1,19, Rick Quax20
* Shared first author

**Affiliations:**^1^Unit of Medical Technology and Intelligent Information Systems, Department of Materials Science and Engineering, University of Ioannina, Ioannina, Greece.
^2^Department of Biological Applications and Technology, University of Ioannina, Ioannina, Greece.
^3^Department of Clinical Psychology, University of Amsterdam, Amsterdam, The Netherlands.
^4^Department of Psychiatry, Amsterdam UMC location Vrije Universiteit Amsterdam, Boelelaan 1117, Amsterdam, The Netherlands.
^5^Amsterdam Public Health, Mental Health Program, Amsterdam The Netherlands.
^6^Amsterdam Public Health, Methodology Program, Amsterdam, The Netherlands.
^7^Amsterdam Neuroscience, Mood, Anxiety, Psychosis, Sleep & Stress program, Amsterdam, The Netherlands.
^8^Amsterdam Neuroscience, Complex Trait Genetics, Amsterdam, The Netherlands.
^9^Department of medical Psychology, Amsterdam University Medical Centers, Amsterdam, The Netherlands.
^10^Central Diagnostic Laboratory, Division Laboratories, Pharmacy, and Biomedical genetics, University Medical Center Utrecht, Utrecht University, the Netherlands.
^11^Department of Genomic Sciences, University of Virginia, Charlottesville, VA, USA.
^12^Department of Clinical Chemistry, Faculty of Medicine and Health Technology, Tampere University, Tampere, Finland.
13Faculty of Medicine and Health Technology, Finnish Cardiovascular Research Center Tampere, Tampere University, Tampere, Finland.
^14^Department of Clinical Chemistry, Fimlab Laboratories, Tampere, Finland.
^15^Department of Clinical Physiology, Tampere University Hospital, Tampere Finland.
^16^Research Centre of Applied and Preventive Cardiovascular Medicine, University of Turku, Turku, Finland.
^17^Department of Clinical Physiology and Nuclear Medicine, Turku University Hospital, Turku, Finland.
^18^Centre for Population Health Research, University of Turku and Turku University Hospital, Turku, Finland.
^19^Biomedical Research Institute, Foundation for Research and Technology - Hellas (FORTH), Ioannina, Greece.
^20^Computational Science Lab, Institute of Informatics, University of Amsterdam, Amsterdam, The Netherlands.

**Correspondence address:**

[**a.koloi@uva.nl**](mailto:a.koloi@uva.nl)**,** Valschermkade 19, 1059 CD Amsterdam, The Netherlands.

[**a.o.rydin@amsterdamumc.nl**](mailto:a.o.rydin@amsterdamumc.nl)**,** Oldenaller 1, 1081 HJ Amsterdam, The Netherlands.

**ORCID digits:** 0009-0001-8197-3749, 0000-0002-5525-3350, 0000-0002-3697-6617, 0000-0003-4344-5766, 0000-0001-7780-4806, 0009-0001-9702-4185, 0000-0001-6888-1404, 0000-0001-5177-3431, 0000-0002-2555-4424, 0000-0002-4510-7341, 0000-0001-5905-1206, 0000-0001-9365-3702, 0000-0002-7362-5082, 0000-0002-0299-0074

# Introduction

This document contributes as supplemental information regarding the work “Morbidity-bridging metabolic pathways: linking early cardiovascular disease risk and depression symptoms using a multi-modal approach”. This work is divided into two sections: Supplemental Materials, Supplemental Methods, Supplemental Results, Supplemental Figures and Supplemental Tables. In the Supplemental Materials, we provide more detailed information on the variables selected in the Young Finns Study (YFS) and UK Biobank (UKB). In the Supplemental Methods section, we elaborate on the first part of the analysis, namely the network analysis. This is followed by further explanation regarding the third part of the analysis: the Mendelian randomization (MR).

# Supplemental Materials

## Study cohorts

For our work, we make use of two cohort studies: the YFS serves as input data for the network analysis step, and UKB provides data for the external validation. In the main text we describe the variables, which are discussed in more details below.

### Young Finns study

The present analyses drew from the Cardiovascular Risk in YFS, a population-based prospective cohort study carried out at five medical schools in Finland (Turku, Helsinki, Kuopio, Tampere, and Oulu). The YFS aimed to thoroughly evaluate cardiovascular risk factors in children and adolescents across the nation. The study began in 1980, and in 2007 2,200 individuals aged 30-45 years old took part in the 27-year follow-up. The study design received approval from the ethical committees of all Finnish universities with medical faculties, including those at Helsinki, Turku, Tampere, Kuopio, and Oulu. Informed consent was obtained from all participants, or their parents if participants were under 18 years old, prior to participation. The study adhered to the principles outlined in the Declaration of Helsinki. The design of the YFS is described with further details elsewhere.^1^

The study did not screen the entire population but used a representative sample from the YFS cohort, which is a longitudinal study tracking participants over 40 years. In this study, the inclusion criteria were defined to ensure comprehensive data availability for each participant. Specifically, individuals were included in the analysis if they had complete data for the following key variables:

1. Beck's Depression Inventory (BDI), providing a measure of depressive symptoms.
2. CVD risk factors, including metrics such as blood pressure, carotid intima-media thickness (cIMT).
3. Plasma metabolite profiles obtained through NMR spectroscopy.

#### Metabolites

Assessments were based on a proton nuclear magnetic resonance (NMR) platform provided by Nightingale Health Ltd. in Helsinki, Finland, to quantify concentrations of various lipid-related metabolites and their ratios.^2^ Among the measures provided by the platform, we selected concentrations of 52 lipids, fatty acids, and low molecular weight metabolites as in a previous study.^3^ The metabolites are categorised into eleven distinct groups, encompassing amino acids, apolipoproteins, cholesterol, fatty acids, fluid balance, glycerides and phospholipids, glycolysis-related metabolites, inflammation markers, ketone bodies, lipoprotein particle size, and fatty acids measures (**Supplemental Table 1**).

#### Depression symptoms

These items encompassed a range of somatic and cognitive experiences related to depression, including feelings of sadness, pessimism, past failure, loss of pleasure, guilty feelings, punishment feelings, self-dislike, self-criticalness, suicidal thoughts or wishes, crying, agitation, loss of interest, indecisiveness, worthlessness, loss of energy, changes in sleep pattern, irritability, changes in appetite, difficulty concentrating, tiredness or fatigue, and loss of interest in sex. Each of the answers are scored on a Likert scale value of 0 to 3 with higher scores representing higher levels of depression, except for changes in sleep/appetite: these variables had a score of 0 to 6 (**Supplemental Table 2**).

#### CVD risk factors

We selected a set of three CVD risk factors (**Supplemental Table 3** for details), systolic and diastolic blood pressure and cIMT as cardiovascular risk factors because they provide robust indicators of cardiovascular risk. In the Young Finns Study in 2007, the assessment of carotid intima-media thickness (IMT), systolic blood pressure (SBP), and diastolic blood pressure (DBP) was conducted as follows: cIMT was assessed using high-resolution B-mode ultrasonography of the carotid arteries. Thisnon-invasive imaging technique precisely measured the combined thickness of the intima and media layers of the carotid artery wall. The cIMT measurement serves as a validated surrogate marker for subclinical atherosclerosis and is widely recognized as an early indicator of cardiovascular risk.^1,41,4^ Blood pressure was measured using an Arteriosonde 1020 (Roche) ultrasound device and a standard mercury sphygmomanometer. Participants rested seated for 5 minutes, with measurements taken on the right arm. At least three readings were performed, recorded to the nearest even millimetre of mercury, with the mean value representing the participant's blood pressure.^1^ Other CVD indicators, available in YFS, were already represented within our metabolite data. Additionally, variables like smoking and alcohol consumption, physical activity were treated as covariates rather than primary variables of interest.

### UK biobank

The UK Biobank is a population-based cohort comprising 502,524 UK residents aged 40-69 years who were recruited between 2006 and 2010 from 22 assessment centres throughout the UK to reflect a broad socioeconomic demographic and mixture of urban and rural residents.^5^ The full dataset includes a range of phenotyping assessments, biochemical assays and genome-wide genotyping of baseline samples from all participants. The UK Biobank study was approved by the UK Biobank’s research ethics committee and Human Tissue Authority research tissue bank. Informed consent was obtained from all participants.

UKB provides a robust dataset for external validation of YFS findings, featuring key variables such as metabolic biomarkers quantified using the same NMR platform as YFS. Although there is a significant age gap between YFS and UKB, several key risk factors show similarities between the cohorts. Hypertension and BMI are reportedly comparable, suggesting consistency in these measures across different life stages. Smoking status, while not directly equivalent, shows potentially overlapping trends: 19% of YFS participants report positive smoking status, while UKB data indicates 8% current smokers and 36% former smokers. Although age stratification could be a valuable approach to implement, it is important to note that there is no age overlap between the cohorts at the specific time points used. This lack of overlap limits direct comparisons within age groups but allows for the examination of age-dependent trends in cardiometabolic risk factors.

#### Metabolites and CVD risk factors

The NMR dataset included detailed metabolic biomarkers, quantified from 118,461 baseline plasma samples, processed by Nightingale Health Plc. This large-scale dataset represents a substantial improvement over previous metabolic profiling studies, with a sample size more than ten times larger than many earlier efforts. In addition, key CVD risk factors, such as cIMT (data field [22671](https://biobank.ndph.ox.ac.uk/showcase/field.cgi?id=22671)) and diastolic blood pressure (data field [4079](https://biobank.ndph.ox.ac.uk/showcase/field.cgi?id=4079)) were assessed in the UKB. cIMT was measured using the CardioHealth Station ultrasound system. Assessments were performed at four angles following predefined standards. The average value of IMT from the four angles was used as the primary outcome measure.^6^ Blood pressure measurements were conducted using a standardized protocol with an Omron HEM-7015IT digital blood pressure monitor. Participants were seated with feet flat on the floor and any restrictive clothing on the left upper arm removed. After a 5-minute rest period, two measurements were taken on the left arm (right arm if left was impractical), with at least a 1-minute interval between readings. The final blood pressure value was calculated as the mean of these two readings, or the single value was used if only one reading was available. SBP and DBP values were adjusted for medication use by adding 15 mm Hg and 10 mm Hg respectively for individuals taking blood pressure-lowering medication.^7^

While there are some differences in the protocols for assessing CVD risk factors between the YFS and UKB the overall approaches are quite similar and allow for reasonable comparability of results. Both studies use ultrasound techniques to measure cIMT, a validated surrogate marker for subclinical atherosclerosis, with YFS using high-resolution B-mode ultrasonography and UKB using the CardioHealth Station system. For blood pressure measurements, both studies follow similar protocols: participants are seated and rested before measurements, multiple readings are taken, and the mean is used as the final value. The main differences in blood pressure measurement (YFS using Arteriosonde 1020 and mercury sphygmomanometer on the right arm, UKB using an Omron digital monitor on the left arm) are minor and unlikely to significantly impact result comparability. In conclusion, while some protocol differences exist, they are not substantial enough to prevent meaningful comparisons between the two cohorts.

#### Covariates

In the UK Biobank, covariates were measured during the baseline assessment. Age and sex were recorded as basic sociodemographic characteristics. Smoking status was self-reported as "never", "previous", or "current". Physical activity was initially assessed based on the duration of moderate physical activity and subsequently categorized into a binary variable, indicating whether the participant engaged in moderate physical activity or did not (**Supplemental Table 4**). Further information is available online from relevant UK Biobank data fields per covariate; sex (data field [31](https://biobank.ctsu.ox.ac.uk/crystal/field.cgi?id=31)), age (data field [21003](https://biobank.ctsu.ox.ac.uk/crystal/field.cgi?id=21003)), smoking (data field [20116](https://biobank.ctsu.ox.ac.uk/crystal/field.cgi?id=20116)), physical activity (data field [10962](https://biobank.ndph.ox.ac.uk/showcase/field.cgi?id=10962)).

#### Depression symptoms

This study involved up to 157,286 participants with available data on depression and anxiety symptoms, with the sample size varying depending on the specific symptom assessed (**Supplemental Table 5**). These symptoms were evaluated as part of a follow-up mental health survey in the UK Biobank study. The survey utilized the Patient Health Questionnaire-9 (PHQ-9)^8^ to assess nine depression symptoms and the General Anxiety Disorder-7 (GAD-7)^9^ to evaluate seven anxiety symptoms.

YFS and UKB both assessed depressive symptoms, not formal diagnoses, using the BDI-II and PHQ-9. These tools measure symptom severity based on diagnostic criteria, covering core symptoms like including mood, sleep disturbances, appetite changes, and feelings of worthlessness. Established score thresholds can identify likely depression cases; for example, a PHQ-9 score ≥10 indicates likely major depression. While not diagnostic, these measures allow researchers to define groups with clinically significant depression symptoms.

The existing literature demonstrates a robust relationship between PHQ-9 and BDI-II scores. A study by Kung et al. (2013) found an overall correlation of r=0.77 between PHQ-9 and BDI-II scores, indicating a strong correlation.^10^ Another study found a good correlation between BDI-II and PHQ-9, with a correlation coefficient (r) of 0.74.^11^ A study by Weobong et al. (2018) reported high correlations between PHQ-9 and BDI-II across different populations: India (r=0.79), UK (r=0.87), and US (r=0.77).^12^ Findings suggest that both instruments capture similar constructs of depressive symptoms, despite differences in their specific content, and provide a strong rationale for the comparability of results.

# Supplemental Methods

This section presents elaborations on certain aspects of the methods section. The approach constituted of 3 analyses: (i) a network analysis following the leveraging of a network; (ii) external robustness check of the findings through an Ordinary Least Squares (OLS) Regression, and (iii) Mendelian randomization analysis checking for mechanistic relations found by steps (i) and (ii). The main text explains the overview of these steps; here we go into more detail regarding the justification and mathematical background of the analysis regarding steps (i) and (iii).

## Network analysis

In this section we describe the mathematical assumptions for Mixed Graphical Models, the method that was used to create a network of the YFS data of depression symptoms, CVD risk factors and metabolites. This network was analyzed on stability and centrality. Both approaches are described in further detail below.

### Mixed Graphical models

Each variable (76 in total) was treated as a random variable, and their joint distribution was assumed to be factorized according to an undirected graph G, following the principles of the Global Markov Property. The MGM approach formalized the connection between the (multivariate) probability distribution and the graph structure. The variables, represented as nodes in the graph, were interconnected through conditional distributions, which belonged to the exponential family. The parameters associated with these distributions served as the edge weights within the network, capturing the strength of the associations between the variables. To enhance the reliability and robustness of our findings, we employed 10-fold cross-validation as a penalty parameter. In k-fold cross-validation, the data is divided into k equally sized folds, and the model is trained on k-1 folds while the remaining fold is used for validation. This process is repeated k times, with each fold being used exactly once as the validation data. The results are averaged to estimate the model's performance. This approach effectively prevented overfitting and improved the generalizability of our results. By incorporating cross-validation, we accounted for the intricacies and complexities of the data, thereby reducing the risk of drawing spurious associations. This ensured that the associations identified in our analysis were more reliable, as they were validated on unseen data.

### Stability analysis

We performed stability analyses through bootstrapping, adhering to the methodology described in prior research studies.^13,14^ This involved running the model 100 times for each potential edge in the network, resulting in an edge-stability measure. We specifically focused on metabolites that were both connected to depression symptoms and the CVD risk factors, disregarding any connections between other metabolites. For these selected edges, we calculated the 95% confidence interval, representing the range between the 2.5th and 97.5th percentiles, as well as the mean of the edge strengths. These measures provided insights into the variation and central tendencies of the edge strengths for the identified associations. Furthermore, we computed the fraction of times that each edge appeared in the bootstraps. This fraction served as an additional measure of edge-stability, indicating the frequency at which the edge was present among the resampled datasets.

To further evaluate the robustness of the associations between depressive symptoms, CVD risk factors, and metabolites, a permutation test with 1,000 iterations was conducted. Partial correlations for each symptom-metabolite and CVD risk factor-metabolite pair were first computed using the original data. To test the null hypothesis of no association, symptom and CVD risk values were randomly shuffled across samples while keeping metabolite values unchanged.^15^ Partial correlations were then recalculated for each permutation, generating a null distribution of correlation values. A permutation-based p- value (pperm​) was determined by calculating the proportion of permuted correlations with an absolute value equal to or greater than the observed correlation. Pairs with pperm​ < .05 were considered statistically significant. This method confirmed that the observed associations were highly unlikely to be due to chance, reinforcing their reliability.

### Centrality and jointness (comorbidity) assessment

Degree centrality sums the edge weights of a node; we considered nodes with a relatively lower degree centrality to be more influential on the system: in the case of an edge disappearing, the node will be affected more strongly because it is not “supported” as much by other nodes.

The jointness score is a measure summing to evaluate the collective significance of common metabolites factors in relation to both CVD risk factors and depression symptoms. To achieve this, we introduced a metric termed the Jointness score. This score is calculated using a defined formula aimed at capturing the combined effect of various metabolites on both phenotypes. The formula for the Jointness score is expressed as follows:

$$Jointness Score\left( {metabolite}_{i} \right):=$$

$$\sum jweight\left[ {metabolite}_{i}, CVD risk factors \right]\times\sum kweight\left[ {metabolite}_{i}, Depressive symptoms \right]$$

Here, ${metabolite}_{i}$ signifies the specific metabolite under investigation. The Jointness Score calculation involved summing the weights associated with the relationships between the chosen metabolite and CVD risk factors, followed by multiplication with the sum of weights pertaining to the associations with depression symptom variables.

This formula is designed to yield a relatively high score for metabolites that have significant weights to both morbidities, and low score to metabolites that are only connected to one of the morbidities. That is, if the sum of edge weights to one (or both) of the morbidities is (close to) zero, then the result of the multiplication will be (close to) zero. Only if a metabolite has some non-zero edge weights to each of the two morbidities, will the result of the multiplication be non-zero. Of these shared metabolites, the stronger the edge weights, the higher the jointness score. The absolute value of the jointness score of a particular metabolite is not meant to be meaningful; only its relative value to other metabolites is deemed meaningful.

## Mendelian randomization

Mendelian randomization (MR) is a research method employing genomics used to infer causal relationships between risk factors and health outcomes. Genomics provide opportunities from large genome-wide association studies (GWAS).^16^ The approach is based on the principle that genetic variants are randomly assigned at conception, similar to the randomization process in controlled clinical trials. This randomness minimizes the potential for confounding factors, which often obscure causal inference in observational studies.^17^ MR uniquely utilizes genetic variants as Instrumental Variables (IVs) to assess whether an exposure, such as a biomarker or lifestyle factor, has a direct causal effect on a disease or condition. This approach has become a powerful tool in epidemiology for understanding the underlying mechanisms of complex diseases.

The CVD phenotypes included: Stroke (ALLSTROKE); Ischemic Stroke (IS); Cardio-Embolic Stroke (CES); Large Artery Stroke (LAS); Small Vessel Disease (SVD); Coronary Artery Disease (CAD); Coronary Artery Calcification (CAC), and Carotid Intima Media Thickness (CIMT).^18–21^ Summary statistics for depression were obtained from a meta-analysis of three large genome-wide association studies, encompassing data from 807,553 individuals (246,363 cases and 561,190 controls), which identified 102 independent variants, 269 genes, and 15 gene sets associated with the disorder.^22^ Summary statistics for the selected metabolites came from a GWAS^23^ using the Nightingale Health high-throughput metabolomics platform. These platforms have provided significant insights into human metabolism, elucidating genetic determinants and their roles in biological mechanisms and complex diseases. GWAS dataset comprising 233 circulating metabolic traits quantified by nuclear magnetic resonance spectroscopy. This dataset includes data from up to 136,016 participants across 33 cohorts, enabling robust analyses of genetic associations. (see **Supplemental Table 6** for sample size and references). The first step was to pre-process the GWAS data of the phenotype selected as exposure, where only Single Nucleotide Polymorphisms (SNPs) present in the 1000 Genomes European reference *panel^24^* were retained, and reference SNP ID (rsIDs) were assigned. Additionally, we applied filtering criteria, keeping SNPs with minor allele frequency (MAF) > 0.01 and p-value < 5e-8. Clumping was performed using the 1000 Genomes European ancestry linkage disequilibrium (LD) reference with a window size of 10,000 kb and an R² threshold of 0.001 (MR default).

In MR analyses several sensitivity analysis steps are required in order to assess whether explanations other than causality may apply. The first step is to measure the strength of the Instrumental Variable (IV) in the study, for which the F-statistic is used. F-statistics<10 suggest that the genetic variants used as IVs are not associated sufficiently with the exposure. The second part of the sensitivity analysis is to compare the main causal effects of the IVW with the MR-Egger and Weighted-median (WM) estimates. These estimates have stricter assumptions. Another assumption that requires testing is the absence or presence of heterogeneity: if the instruments are invalid, they will provide inconsistent estimates. Heterogeneity is tested through Cochran’s Q test: significant estimates > 0 imply evidence for heterogeneity (which may indicate pleiotropy). Lastly, horizontal pleiotropy is tested for. Horizontal pleiotropy happens when an instrument (genetic variant) influences the outcome through pathways other than the exposure.

## Tools and packages

The analyses were conducted using R version 4.1.3, employing several key packages: mgm (v1.2-12) for mixed graphical model implementation, qgraph (v1.9.4) for network analysis and visualization, TwoSampleMR (v0.5.6) for Mendelian randomization analyses, ggplot2 (v3.3.5) for data visualization, and dplyr (v1.0.7) for data manipulation. Additional software utilized included Gephi (v0.10) for advanced network visualization and Python (v3.11.4) with scikit-learn for data preprocessing and multiple imputation.

# Supplemental Results

## Mendelian Randomization findings

In **Supplemental Table 10** we see the ORs (and 95% CI) of the exposures and outcomes, the number of SNPs and the p-value, with three different MR methods. The IVW showed significant (p<0.05) connections between glucose and IS, glucose and CAD, Omega-3 FAs and CAD, Omega-3 FAs and CAC. The WM and MR Egger method showed significant results (p-values below 0.05) for all above-mentioned connections, except glucose and IS, so these connections might be due to (directional) pleiotropy. Additionally, **Supplemental** **Table 11** shows Glucose-CAD, and Omega-3 FAs have high Q statistics with p-values below significance levels. This indicates evidence of heterogeneity. Lastly. **Supplemental Table 12** shows that Glucose and CAD may be influenced by directional pleiotropy (p=0.04); the other two connections, Omega-3 FAs, and CAD/CAC show no evidence of pleiotropic effects.

# Supplemental Figures


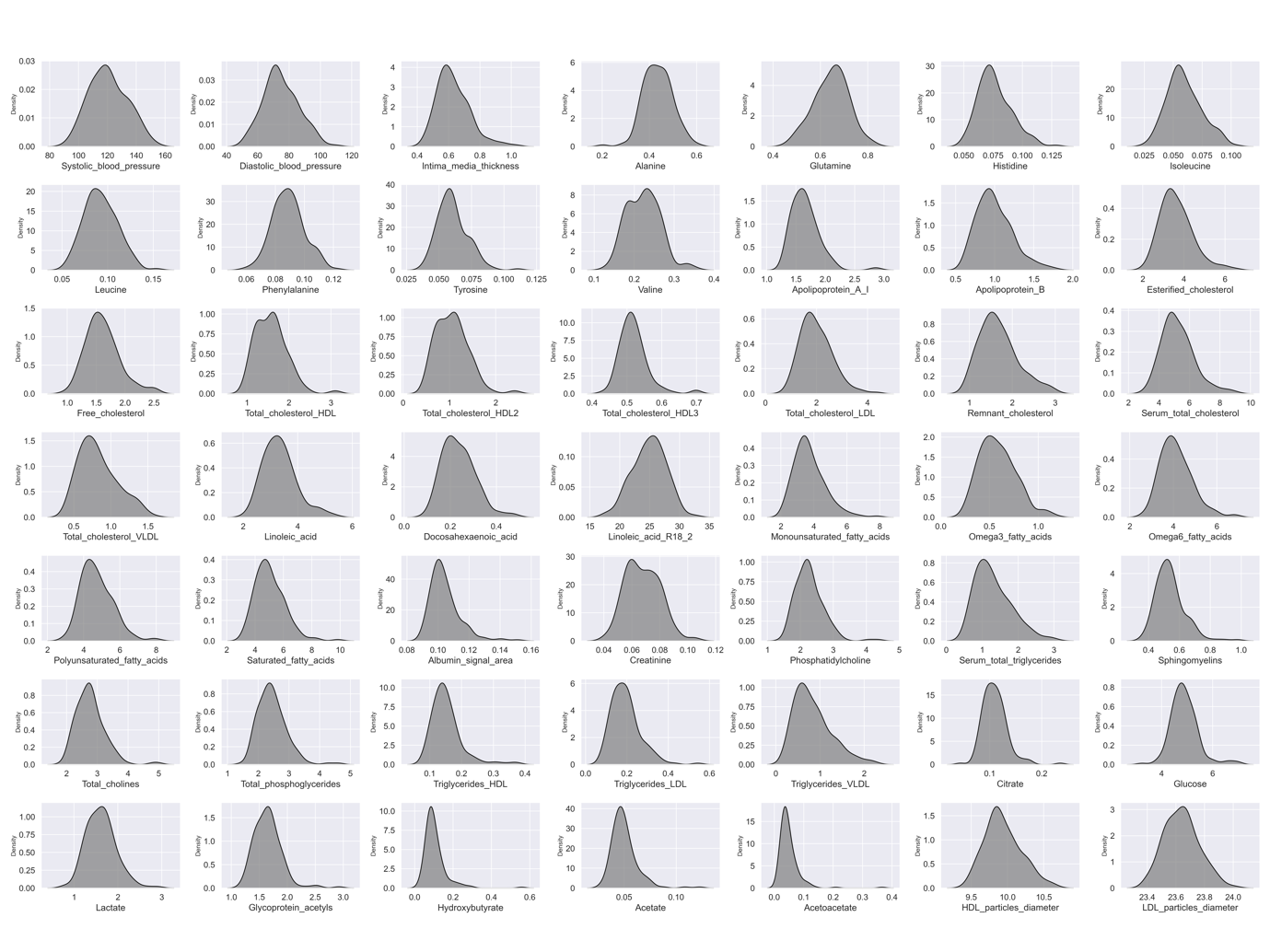


**Supplemental Figure 1**. Metabolites distribution.


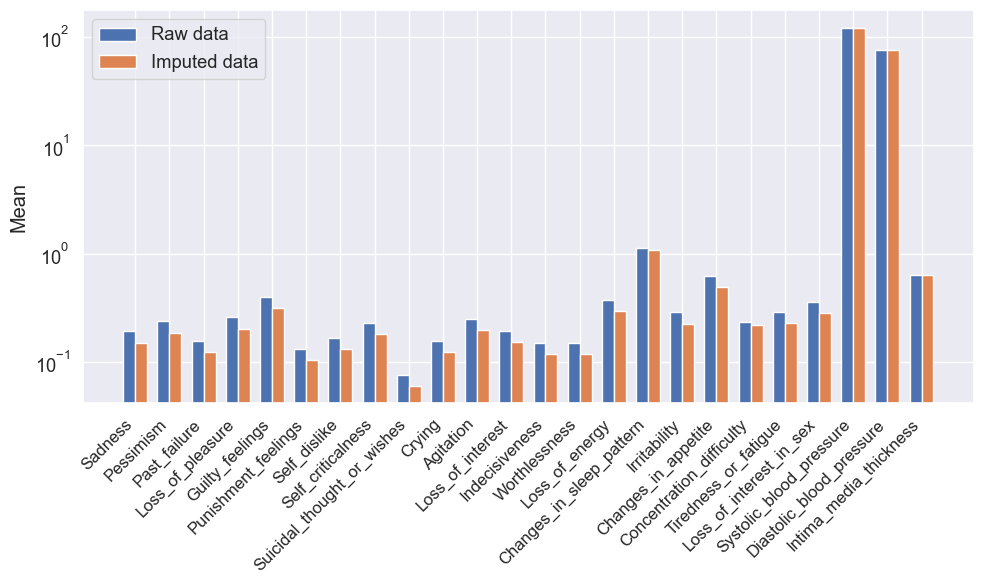

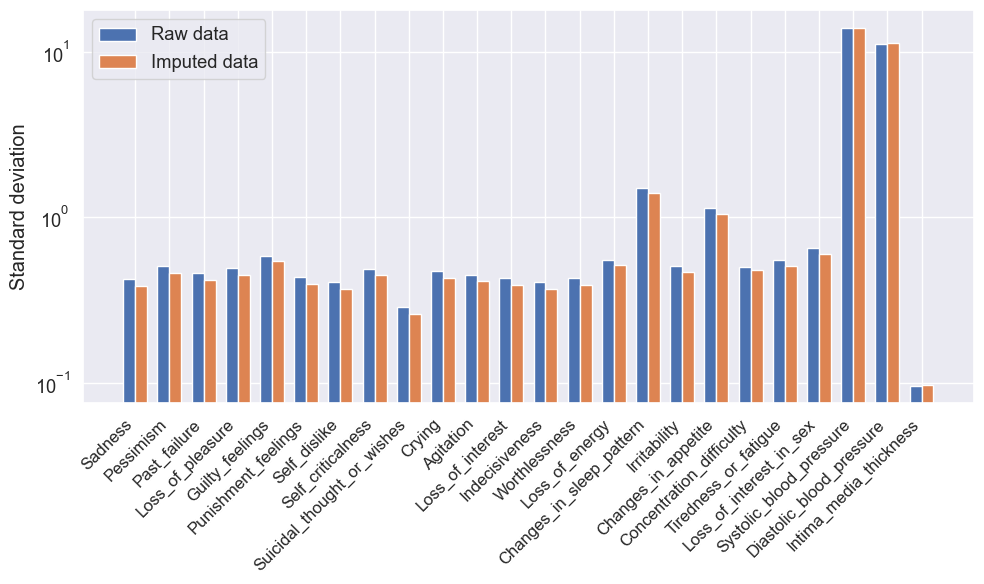


Figure S.

**Supplemental Figure 2.** Comparison of mean and standard deviation statistics before and after imputation.


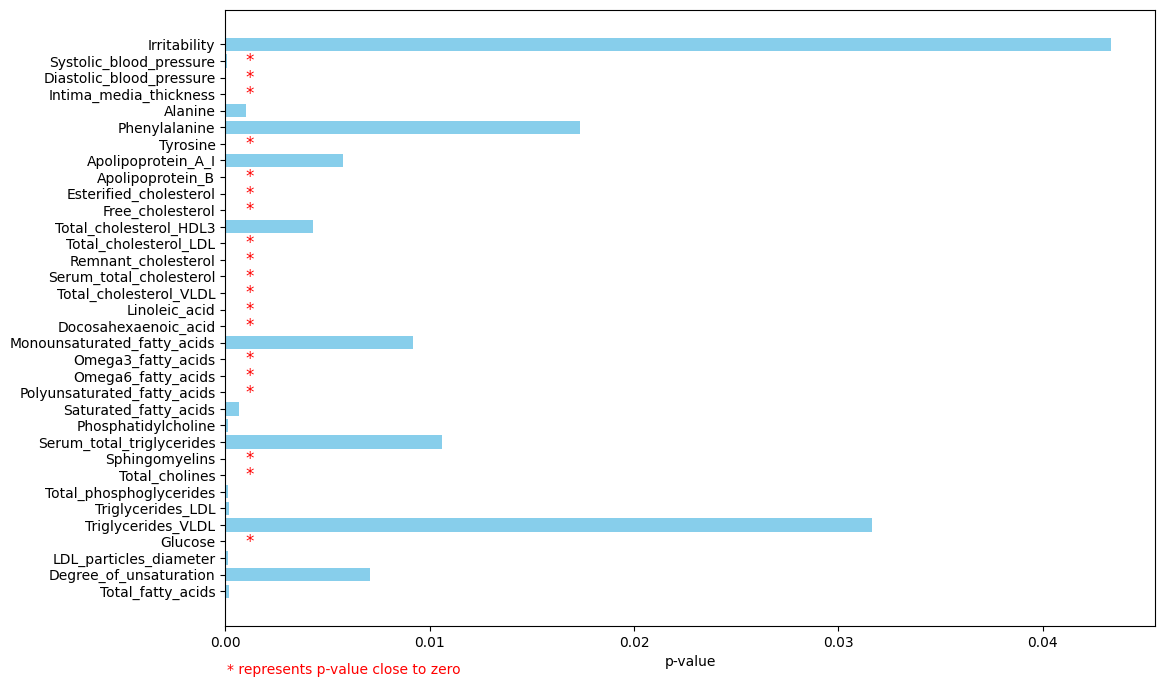

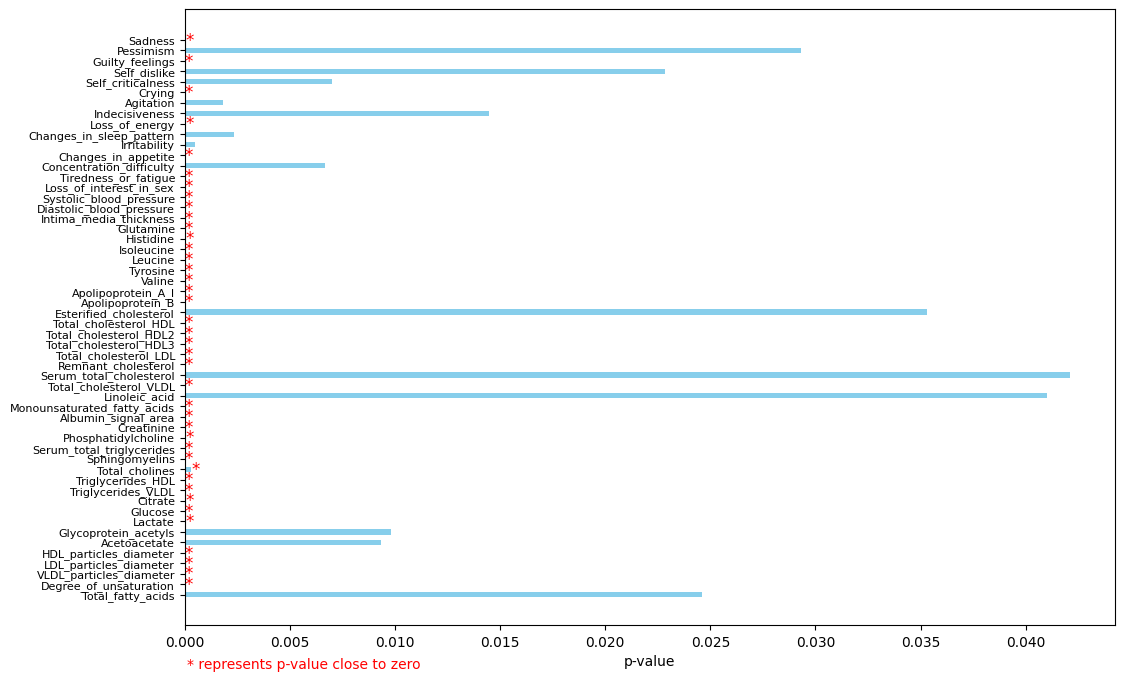


**Supplemental Figure 3**. ANOVA results for the significance of depression symptoms, metabolites, and CVD risk factors in relation to age and gender. The top plot represents the associations with age, while the bottom plot illustrates the associations with gender.

# Supplemental Tables

**Supplemental Table 1.** Metabolites and their grouping.

| Metabolite category | Metabolite (unit) |
| --- | --- |
| Amino acids | Alanine (mmol/L) |
|  | Glutamine (mmol/L) |
|  | Histidine (mmol/L) |
|  | Isoleucine (mmol/L) |
|  | Leucine (mmol/L) |
|  | Phenylalanine (mmol/L) |
|  | Tyrosine (mmol/L) |
|  | Valine (mmol/L) |
| Apolipoproteins | Apolipoprotein A-I (g/L) |
|  | Apolipoprotein B (g/L) |
| Cholesterol | Esterified cholesterol (mmol/L) |
|  | Free cholesterol (mmol/L) |
|  | HDL cholesterol (mmol/L) |
|  | HDL2 cholesterol (mmol/L) |
|  | HDL3 cholesterol (mmol/L) |
|  | LDL cholesterol (mmol/L) |
|  | Remnant cholesterol (mmol/L) |
|  | Serum total cholesterol (mmol/L) |
|  | VLDL cholesterol (mmol/L) |
| Fatty acids | Conjugated linoleic acids (mmol/L) |
|  | Docosahexaenoic acid, 22:6 (mmol/L) |
|  | Linoleic acid, 18:2 (mmol/L) |
|  | Monounsaturated fatty acids (mmol/L) |
|  | Omega-3 fatty acids (mmol/L) |
|  | Omega-6 fatty acids (mmol/L) |
|  | Polyunsaturated fatty acids (mmol/L) |
|  | Saturated fatty acids (mmol/L) |
| Fluid balance | Albumin (signal area) |
|  | Creatinine (mmol/L) |
| Glycerides and phospholipids | Phosphatidylcholine and other cholines (mmol/L) |
|  | Serum total triglycerides (mmol/L) |
|  | Sphingomyelins (mmol/L) |
|  | Total cholines (mmol/L) |
|  | Total phosphoglycerides (mmol/L) |
|  | Triglycerides in HDL (mmol/L) |
|  | Triglycerides in LDL (mmol/L) |
|  | Triglycerides in VLDL (mmol/L) |
| Glycolysis related metabolites | Citrate (mmol/L) |
|  | Glucose (mmol/L) |
|  | Lactate (mmol/L) |
| Inflammation | Glycoprotein acetyls (mmol/L) |
| Ketone bodies | 3-hydroxybutyrate (mmol/L) |
|  | Acetate (mmol/L) |
|  | Acetoacetate (mmol/L) |
| Lipoprotein particle size | Mean diameter for HDL particles (nm) |
|  | Mean diameter for LDL particles (nm) |
|  | Mean diameter for VLDL particles (nm) |
| Total fatty acids and saturation measures | Estimated degree of unsaturation |
|  | Estimated description of fatty acid chain length[ΑΚ2] |
|  | Total fatty acids (mmol/L) |

**Supplemental Table 2.** Extensive data on averages of symptoms from YFS.

| Symptom | Age group | 0 | 1 | 2 | 3 | 4 | 5 | 6 |
| --- | --- | --- | --- | --- | --- | --- | --- | --- |
| Agitation | 30-37 | 435 | 146 | 7 | 0 | 0 |  |  |
|  | 38-45 | 518 | 155 | 0 | 0 | 0 |  |  |
| Changes in appetite | 30-37 | 399 | 71 | 92 | 5 | 9 | 0 | 12 |
|  | 38-45 | 456 | 92 | 91 | 9 | 15 | 2 | 7 |
| Changes in sleep pattern | 30-37 | 278 | 90 | 164 | 13 | 25 | 2 | 16 |
|  | 38-45 | 351 | 93 | 138 | 31 | 19 | 2 | 35 |
| Concentration difficulty | 30-37 | 479 | 91 | 16 | 2 | 0 |  |  |
|  | 38-45 | 532 | 116 | 24 | 0 | 0 |  |  |
| Crying | 30-37 | 512 | 67 | 4 | 5 | 0 |  |  |
|  | 38-45 | 596 | 60 | 5 | 12 | 0 |  |  |
| Guilty feelings | 30-37 | 379 | 191 | 14 | 4 | 0 |  |  |
|  | 38-45 | 428 | 224 | 15 | 7 | 0 |  |  |
| Indecisiveness | 30-37 | 515 | 60 | 12 | 0 | 0 |  |  |
|  | 38-45 | 579 | 81 | 12 | 0 | 0 |  |  |
| Irritability | 30-37 | 414 | 160 | 12 | 1 | 0 |  |  |
|  | 38-45 | 511 | 147 | 11 | 2 | 0 |  |  |
| Loss of energy | 30-37 | 398 | 173 | 15 | 1 | 0 |  |  |
|  | 38-45 | 426 | 221 | 22 | 1 | 0 |  |  |
| Loss of interest | 30-37 | 492 | 93 | 3 | 0 | 0 |  |  |
|  | 38-45 | 539 | 126 | 5 | 3 | 0 |  |  |
| Loss of interest in sex | 30-37 | 437 | 119 | 20 | 11 | 0 |  |  |
|  | 38-45 | 471 | 154 | 32 | 13 | 0 |  |  |
| Loss of pleasure | 30-37 | 458 | 118 | 8 | 4 | 0 |  |  |
|  | 38-45 | 503 | 165 | 3 | 3 | 0 |  |  |
| Past failure | 30-37 | 515 | 60 | 11 | 2 | 0 |  |  |
|  | 38-45 | 593 | 57 | 19 | 5 | 0 |  |  |
| Pessimism | 30-37 | 464 | 110 | 9 | 5 | 0 |  |  |
|  | 38-45 | 540 | 115 | 16 | 3 | 0 |  |  |
| Punishment feelings | 30-37 | 531 | 42 | 12 | 2 | 0 |  |  |
|  | 38-45 | 601 | 53 | 12 | 6 | 0 |  |  |
| Sadness | 30-37 | 481 | 100 | 6 | 1 | 0 |  |  |
|  | 38-45 | 552 | 118 | 3 | 1 | 0 |  |  |
| Self-criticalness | 30-37 | 461 | 110 | 16 | 1 | 0 |  |  |
|  | 38-45 | 545 | 112 | 14 | 2 | 0 |  |  |
| Self-dislike | 30-37 | 491 | 91 | 5 | 1 | 0 |  |  |
|  | 38-45 | 575 | 92 | 5 | 1 | 0 |  |  |
| Suicidal thought or wishes | 30-37 | 546 | 38 | 3 | 1 | 0 |  |  |
|  | 38-45 | 625 | 45 | 2 | 0 | 0 |  |  |
| Tiredness or fatigue | 30-37 | 447 | 111 | 28 | 1 | 0 |  |  |
|  | 38-45 | 507 | 140 | 22 | 3 | 0 |  |  |
| Worthlessness | 30-37 | 518 | 56 | 12 | 2 | 0 |  |  |
|  | 38-45 | 583 | 79 | 6 | 4 | 0 |  |  |

**Supplemental Table 3.** More extensive data CVD risk factors from YFS.

| Age group | Systolic blood pressure | Diastolic blood pressure | Intima media thickness |
| --- | --- | --- | --- |
| 30-37 | 119.72±12.94 | 74.07±11.06 | 0.60±0.08 |
| 38-45 | 122.43±14.69 | 77.68±11.01 | 0.66±0.10 |

**Supplemental Table 4.** Data characteristics of UK Biobank.

| **Characteristics** | N= 69,513 |
| --- | --- |
|  |  |
| *Sociodemographic* |  |
| **Sex (F)** *(%)* | 64 |
| **Age** *years (mean ± SD)* | 63.08 ± 7.68 |
| *Health Indicators* |  |
| **BMI** *(mean ± SD)* | 26.92 ± 4.78 |
| **Smokers** *(%)* |  |
| never smoked | 55.58 |
| previous smoker | 35.92 |
| current smoker | 8.49 |
| **Moderate Physical Activity** *(%)* | 87.65 |
| **CVD** *(%)* | 23.14 |
| **Hypertension** *(%)* | 6.3 |
| **Diabetes** *(%)* | 3.24 |
| **Depressed at Baseline** *(%)* | 29.73 |

**Supplemental Table 5.** More extensive data of symptoms from UK Biobank.

|  | Scale | | | | | |
| --- | --- | --- | --- | --- | --- | --- |
| Symptom | | 0 | 1 | 2 | 3 | 4 |
| Ever thought that life not worth living | | 40617 | 14322 | 20613 | N/A | N/A |
|  |  |  |  |  |  |  |
| Ever had prolonged loss of interest in normal activities | | 21890 | 54130 | N/A | N/A | N/A |
|  |  |  |  |  |  |  |
| Recent lack of interest or pleasure in doing things | | N/A | 54935 | 15930 | 2912 | 2178 |
|  |  |  |  |  |  |  |
| Recent poor appetite or overeating | | N/A | 55903 | 13573 | 3393 | 3203 |
|  |  |  |  |  |  |  |
| Trouble sleeping | | N/A | 32169 | 28531 | 6984 | 8476 |
|  |  |  |  |  |  |  |
| Sleeping change | | 15410 | 60750 | N/A | N/A | N/A |
|  |  |  |  |  |  |  |
| Trouble falling or staying asleep, or sleeping too much | | N/A | 32169 | 28531 | 6984 | 8476 |
|  |  |  |  |  |  |  |
| Sleeplessness / insomnia | | N/A | 16811 | 36465 | 22854 | N/A |
|  |  |  |  |  |  |  |

**Supplemental Table 6.** GWAS references and sample sizes.

| **Trait Number** | **Trait or disease** | **Sample size** | **PMID** | **Reference** | **Journal** | **Ancestry** |
| --- | --- | --- | --- | --- | --- | --- |
| 1 | Coronary Artery Disease | 1,165,690 | 36474045 | Aragam et. al, 2022 | nature genetics | European |
| 3 | Carotid Intima-Media Thickness | 71,128 | 30510157 | Franceschini et al. 2018 | nature communication | Multi ancestry |
| 5 | Cardio- Embolic stroke | 1,245,612 | 36180795 | Mishra et al., 2022 | nature | European |
| 6 | Any stroke | 1,308,460 | 36180795 | Mishra et al., 2022 | nature | European |
| 7 | Any Ischemic stroke | 1,296,908 | 36180795 | Mishra et al., 2022 | nature | European |
| 8 | Small Vessel Disease | 1,241,619 | 36180795 | Mishra et al., 2022 | nature | European |
| 9 | Large Artery Stroke | 1,241,207 | 36180795 | Mishra et al., 2022 | nature | European |
| 34 | Depression | 807,553 | 30718901 | Howard et al., 2019 | nature neuroscience | European |
| 47 | Coronary Artery Calcification | 35,776 | 37770635 | Kavousi et al., 2022 | nature genetics | Multi ancestry |
| 45 | Glucose | 136,016 | 38448586 | Karjalainen MK et al., 2024 | nature | Multi ancestry |
| 12 | Omega-3 fatty acids | 136,016 | 38448586 | Karjalainen MK et al., 2024 | nature | Multi ancestry |

**Supplemental Table 7.** F Statistics. The table includes the number of SNPs used in the analysis and the minimum and maximum F-statistics, indicating the strength and reliability of the genetic instruments used in the Mendelian Randomization analysis.

| Exposure | Outcome | N SNPs | F-statistics min | F-statistics max |
| --- | --- | --- | --- | --- |
| Glucose | PGC depression | 29 | 29.92 | 630.92 |
|  | ALLSTROKE | 31 | 29.92 | 630.92 |
|  | IS | 31 | 29.92 | 630.92 |
|  | CES | 30 | 29.92 | 630.92 |
|  | LAS | 29 | 29.92 | 630.92 |
|  | SVD | 28 | 29.92 | 630.92 |
|  | CAD | 31 | 29.92 | 630.92 |
|  | CAC | 31 | 29.92 | 630.92 |
|  | CIMT | 31 | 29.92 | 630.92 |
| Omega-3 fatty acids | PGC depression | 74 | 30.41 | 1581.99 |
|  | ALLSTROKE | 74 | 30.41 | 1581.98 |
|  | IS | 72 | 30.41 | 1581.98 |
|  | CES | 70 | 30.41 | 1581.98 |
|  | LAS | 66 | 30.41 | 891.02 |
|  | SVD | 67 | 29.91 | 891.02 |
|  | CAD | 75 | 30.41 | 1581.98 |
|  | CAC | 73 | 30.41 | 1581.98 |
|  | CIMT | 73 | 30.41 | 1581.98 |
| Depression | Glucose | 47 | 29.70 | 78.44 |
|  | Omega-3 fatty acids | 47 | 29.78 | 78.44 |

**Supplemental Table 8**. Missing percentages of YFS variables.

| Variable | Missing Percentage |
| --- | --- |
| Alanine | 0.00 |
| Glutamine | 0.00 |
| Histidine | 0.00 |
| Isoleucine | 0.00 |
| Leucine | 0.00 |
| Phenylalanine | 0.00 |
| Tyrosine | 0.00 |
| Valine | 0.00 |
| Apolipoprotein A I | 0.00 |
| Apolipoprotein B | 0.00 |
| Esterified cholesterol | 0.00 |
| Free cholesterol | 0.00 |
| Total cholesterol HDL | 0.00 |
| Total cholesterol HDL2 | 0.00 |
| Total cholesterol HDL3 | 0.00 |
| Total cholesterol LDL | 0.00 |
| Remnant cholesterol | 0.00 |
| Serum total cholesterol | 0.00 |
| Total cholesterol VLDL | 0.00 |
| Linoleic acid | 0.00 |
| Docosahexaenoic acid | 0.00 |
| Linoleic acid R18 2 | 0.00 |
| Monounsaturated fatty acids | 0.00 |
| Omega3 fatty acids | 0.00 |
| Omega6 fatty acids | 0.00 |
| Polyunsaturated fatty acids | 0.00 |
| Saturated fatty acids | 0.00 |
| Albumin signal area | 0.00 |
| Creatinine | 0.00 |
| Phosphatidylcholine | 0.00 |
| Serum total triglycerides | 0.00 |
| Sphingomyelins | 0.00 |
| Total cholines | 0.00 |
| Total phosphoglycerides | 0.00 |
| Triglycerides HDL | 0.00 |
| Triglycerides LDL | 0.00 |
| Triglycerides VLDL | 0.00 |
| Citrate | 0.00 |
| Glucose | 0.00 |
| Lactate | 0.00 |
| Glycoprotein acetyls | 0.00 |
| Hydroxybutyrate | 0.00 |
| Acetate | 0.00 |
| Acetoacetate | 0.00 |
| HDL particles diameter | 0.00 |
| LDL particles diameter | 0.00 |
| VLDL particles diameter | 0.00 |
| Degree of unsaturation | 0.00 |
| Total fatty acids | 0.00 |
| Sadness | 21.08 |
| Pessimism | 21.08 |
| Past failure | 21.08 |
| Loss of pleasure | 21.08 |
| Guilty feelings | 21.08 |
| Punishment feelings | 21.26 |
| Self-dislike | 21.14 |
| Self-criticalness | 21.14 |
| Suicidal thought or wishes | 21.20 |
| Crying | 21.14 |
| Agitation | 21.14 |
| Loss of interest | 21.14 |
| Indecisiveness | 21.26 |
| Worthlessness | 21.20 |
| Loss of energy | 21.39 |
| Changes in sleep pattern | 21.39 |
| Irritability | 21.33 |
| Changes in appetite | 21.20 |
| Concentration difficulty | 21.20 |
| Tiredness or fatigue | 21.26 |
| Loss of interest in sex | 21.39 |
| age group | 0.00 |
| Gender | 0.00 |
| Systolic blood pressure | 0.63 |
| Diastolic blood pressure | 0.75 |
| Intima media thickness | 0.56 |

**Supplemental Table 9.** Selection of significant metabolites through integrated assessments of bootstrap stability, degree centrality and jointness scores, and OLS robustness using UK Biobank.

| Metabolite | Phenotype | Stability | Centrality | Robustness | Cumulative "score" |
| --- | --- | --- | --- | --- | --- |
| Albumin | change in sleep pattern | unstable | medium central | robust | 2/3 |
|  | Intima-media thickness | semi-stable |  | robust |  |
| Glucose | change in sleep pattern | unstable | not central | robust | 1.5/3 |
|  | Intima-media thickness | stable |  | robust |  |
| Creatinine | loss of interest | semi-stable | medium central | mediated by age | 1.5/3 |
|  | change in sleep pattern | stable |  | not robust |  |
|  | Intima-media thickness | unstable |  | not robust |  |
| Omega-3 fatty acids | change in appetite | semi-stable | central | not robust -> due to "change" variable | 2.5/3 |
|  | Intima-media thickness | semi-stable |  | mediated by age |  |
| Citrate | worthlessness | stable | not central | not robust | 1/3 |
|  | diastolic blood pressure | semi-stable |  | robust |  |

**Supplemental Table 10.** MR sensitivity analysis results using three methods: Inverse Variance Weighted (IVW), Weighted Median (WM), and MR Egger. The table shows number of SNPs used as instruments (nrsnps), odds ratios (OR), confidence intervals (lci95, uci95), and p-values, providing insights into potential causal effects. Cardiometabolic traits analyzed include stroke, ischemic stroke (IS), cardioembolic stroke (CES), large artery stroke (LAS), small vessel disease (SVD), coronary artery disease (CAD), coronary artery calcification (CAC), and carotid intima-media thickness (CIMT).

|  |  |  | WM | | | | MR Egger | | | | IVW | | | |
| --- | --- | --- | --- | --- | --- | --- | --- | --- | --- | --- | --- | --- | --- | --- |
| Exposure | Outcome | nrsnps | or | or_lci95 | or_ucl95 | p-value | or | or_lci95 | or_ucl95 | p-value | or | or_lci95 | or_ucl95 | p-value |
| Glucose | Depression | 29 | 1.01 | 0.94 | 1.08 | 0.76 | 1.09 | 0.96 | 1.23 | 0.192 | 0.98 | 0.92 | 1.04 | 0.524 |
|  | allstroke | 31 | 1.06 | 0.95 | 1.17 | 0.30 | 1.05 | 0.87 | 1.27 | 0.62 | 1.09 | 1 | 1.19 | 0.0516 |
|  | IS | 31 | 1.05 | 0.94 | 1.16 | 0.40 | 1.04 | 0.87 | 1.24 | 0.684 | **1.11** | **1.02** | **1.2** | **0.0193** |
|  | CES | 30 | 1.04 | 0.81 | 1.33 | 0.78 | 0.97 | 0.68 | 1.37 | 0.854 | 1.1 | 0.94 | 1.29 | 0.255 |
|  | LAS | 29 | 1.15 | 0.84 | 1.58 | 0.39 | 0.98 | 0.54 | 1.78 | 0.956 | 1.18 | 0.9 | 1.54 | 0.236 |
|  | SVD | 28 | 1.02 | 0.77 | 1.37 | 0.87 | 0.92 | 0.60 | 1.43 | 0.727 | 1.08 | 0.89 | 1.31 | 0.446 |
|  | CAD | 31 | **1.20** | **1.12** | **1.3** | **<0.0001** | **1.36** | **1.13** | **1.62** | **<0.005** | **1.14** | **1.04** | **1.24** | **<0.005** |
|  | CAC | 31 | 1.03 | 0.78 | 1.38 | 0.82 | 1.27 | 0.84 | 1.92 | 0.27 | 1.11 | 0.92 | 1.35 | 0.283 |
|  | CIMT | 31 | 1.01 | 1.00 | 1.02 | 0.14 | 1.01 | 1.00 | 1.03 | 0.127 | 1.01 | 1 | 1.01 | 0.0843 |
| Omega-3 fatty acids | Depression | 74 | 0.96 | 0.95 | 1.02 | 0.341 | 0.97 | 0.93 | 1.01 | 0.14 | 1 | 0.98 | 1.03 | 0.81 |
|  | allstroke | 74 | 0.98 | 0.92 | 1.03 | 0.418 | 0.98 | 0.89 | 1.08 | 0.722 | 1.04 | 0.98 | 1.1 | 0.174 |
|  | IS | 72 | 1.01 | 0.95 | 1.08 | 0.689 | 0.99 | 0.88 | 1.1 | 0.818 | 1.05 | 0.99 | 1.12 | 0.118 |
|  | CES | 70 | 0.95 | 0.82 | 1.1 | 0.489 | 1.02 | 0.83 | 1.25 | 0.854 | 1 | 0.89 | 1.11 | 0.934 |
|  | LAS | 66 | 1.02 | 0.84 | 1.24 | 0.818 | 0.99 | 0.72 | 1.36 | 0.941 | 1.15 | 0.98 | 1.36 | 0.0875 |
|  | SVD | 67 | 1.2 | 1.01 | 1.43 | 0.034 | 1.08 | 0.84 | 1.38 | 0.545 | 1.13 | 1 | 1.28 | 0.0585 |
|  | CAD | 75 | **1.47** | **1.38** | **1.56** | **<0.0001** | **1.53** | **1.3** | **1.8** | **<0.0001** | **1.46** | **1.33** | **1.6** | **<0.0001** |
|  | CAC | 73 | **1.78** | **1.48** | **2.14** | **<0.0001** | **1.85** | **1.46** | **2.33** | **<0.0001** | **1.71** | **1.5** | **1.95** | **<0.0001** |
|  | CIMT | 73 | 1.01 | 1 | 1.01 | 0.12 | 1.02 | 1 | 1.03 | 0.783 | **1.01** | **1** | **1.02** | **0.0303** |
| Depression | Glucose | 47 | 0.96 | 0.89 | 1.02 | 0.10 | 0.83 | 0.62 | 1.10 | 0.20 | 0.99 | 0.94 | 1.05 | 0.827 |
|  | Omega-3 fatty acids | 47 | 0.96 | 0.88 | 1.04 | 0.52 | 1.06 | 0.81 | 1.4 | 0.66 | 0.99 | 0.94 | 1.04 | 0.68 |

**Supplemental Table 11.** Cochran’s Q test results for both MR Egger and IVW methods. The table lists the Q statistics, degrees of freedom (df), and p-values for each method.

|  |  | MR Egger | | | IVW | | |
| --- | --- | --- | --- | --- | --- | --- | --- |
| Exposure | Outcome | Q | df | p value | Q | df | p value |
| Glucose | PGC depression | 54 | 27 | 0.00153 | 61 | 28 | 0.000298 |
|  | ALLSTROKE | 59 | 29 | 0.000784 | 60 | 30 | 0.00103 |
|  | IS | 44 | 29 | 0.0342 | 45 | 30 | 0.0366 |
|  | CES | 23 | 28 | 0.742 | 23 | 29 | 0.756 |
|  | LAS | 44 | 27 | 0.0208 | 45 | 28 | 0.0236 |
|  | SVD | 22 | 26 | 0.708 | 22 | 27 | 0.724 |
|  | CAD | 93 | 29 | 1.46E-08 | 108 | 30 | 1.11E-10 |
|  | CAC | 28 | 29 | 0.506 | 29 | 30 | 0.532 |
|  | CIMT | 38 | 29 | 0.117 | 39 | 30 | 0.121 |
| Omega-3 fatty acids | PGC depression | 97 | 72 | 0.027 | 102 | 73 | 0.0135 |
|  | ALLSTROKE | 173 | 72 | 2.50E-10 | 178 | 73 | 1.06E-10 |
|  | IS | 179 | 70 | 1.76E-11 | 183 | 71 | 7.37E-12 |
|  | CES | 95 | 68 | 0.005 | 95 | 69 | 1.91E-12 |
|  | LAS | 119 | 64 | 3.42E-05 | 122 | 65 | 2.67E-05 |
|  | SVD | 91 | 65 | 0.019 | 91 | 66 | 2.29E-02 |
|  | CAD | 1074 | 73 | 7.41E-178 | 1081 | 74 | 1.03E-178 |
|  | CAC | 113 | 71 | 1.20E-03 | 114 | 72 | 1.27E-03 |
|  | CIMT | 197 | 71 | 1.02E-13 | 201 | 72 | 4.05E-14 |
| Depression | Glucose | 58 | 45 | 0.097 | 60 | 46 | 0.083 |
|  | Omega-3 fatty acids | 60 | 45 | 0.072 | 60 | 46 | 0.08 |

**Supplemental Table 12.** Egger intercept analysis, which is used to detect directional pleiotropy in MR studies. The table lists the Egger intercept values, standard errors (se), and p-values.

| Exposure | Outcome | Egger intercept | se | pval |
| --- | --- | --- | --- | --- |
| Glucose | PGC depression | -0.00525 | 0.00279 | 0.07 |
|  | ALLSTROKE | 0.00197 | 0.00428 | 0.65 |
|  | IS | 0.00314 | 0.00402 | 0.44 |
|  | CES | 0.00609 | 0.00763 | 0.43 |
|  | LAS | 0.0087 | 0.01306 | 0.51 |
|  | SVD | 0.00752 | 0.00967 | 0.44 |
|  | CAD | -0.00868 | 0.00399 | 0.04 |
|  | CAC | -0.00651 | 0.00918 | 0.48 |
|  | CIMT | -0.00032 | 0.00037 | 0.39 |
| Omega-3 fatty acids | PGC depression | 0.00266 | 0.00131 | 0.05 |
|  | ALLSTROKE | 0.00379 | 0.00282 | 0.18 |
|  | IS | 0.00421 | 0.00324 | 0.20 |
|  | CES | -0.00153 | 0.00573 | 0.79 |
|  | LAS | 0.00971 | 0.00867 | 0.27 |
|  | SVD | 0.00299 | 0.00682 | 0.66 |
|  | CAD | -0.00346 | 0.00498 | 0.49 |
|  | CAC | -0.00549 | 0.00689 | 0.43 |
|  | CIMT | -0.004 | 0.00032 | 0.21 |
| Depression | Glucose | 0.00566 | 0.00443 | 0.20 |
|  | Omega-3 fatty acids | -0.00219 | 0.0042 | 0.60 |

# References

1. Raitakari OT, Juonala M, Rönnemaa T, Keltikangas-Järvinen L, Räsänen L, Pietikäinen M, et al. Cohort Profile: The Cardiovascular Risk in Young Finns Study. International Journal of Epidemiology. 2008 Dec 1;37(6):1220–6.

2. Soininen P, Kangas AJ, Würtz P, Suna T, Ala-Korpela M. Quantitative Serum Nuclear Magnetic Resonance Metabolomics in Cardiovascular Epidemiology and Genetics. Circulation: Cardiovascular Genetics. 2015 Feb;8(1):192–206.

3. Metabolomic profiles discriminating anxiety from depression - Kluiver - 2021 - Acta Psychiatrica Scandinavica - Wiley Online Library [Internet]. [cited 2024 Sep 19]. Available from: https://onlinelibrary.wiley.com/doi/full/10.1111/acps.13310

4. West HW, Juonala M, Gall SL, Kähönen M, Laitinen T, Taittonen L, et al. Exposure to Parental Smoking in Childhood Is Associated With Increased Risk of Carotid Atherosclerotic Plaque in Adulthood. Circulation. 2015 Apr 7;131(14):1239–46.

5. UK biobank: an open access resource for identifying the causes of a wide range of complex diseases of middle and old age - PubMed [Internet]. [cited 2024 Sep 19]. Available from: https://pubmed.ncbi.nlm.nih.gov/25826379/

6. Chen ML, Kho PF, Guarischi-Sousa R, Zhou J, Panyard DJ, Azizi Z, et al. Plasma proteomics and carotid intima-media thickness in the UK biobank cohort. Front Cardiovasc Med. 2024 Oct 2;11:1478600.

7. McGrath BP, Kundu P, Daya N, Coresh J, Selvin E, McEvoy JW, et al. Isolated Diastolic Hypertension in the UK Biobank: A Comparison of ACC/AHA and ESC/NICE Guideline Definitions. Hypertension. 2020 Sep;76(3):699–706.

8. Löwe B, Unützer J, Callahan CM, Perkins AJ, Kroenke K. Monitoring depression treatment outcomes with the patient health questionnaire-9. Med Care. 2004 Dec;42(12):1194–201.

9. Spitzer RL, Kroenke K, Williams JBW, Löwe B. A brief measure for assessing generalized anxiety disorder: the GAD-7. Arch Intern Med. 2006 May 22;166(10):1092–7.

10. Kung S, Alarcon RD, Williams MD, Poppe KA, Jo Moore M, Frye MA. Comparing the Beck Depression Inventory-II (BDI-II) and Patient Health Questionnaire (PHQ-9) depression measures in an integrated mood disorders practice. Journal of Affective Disorders. 2013 Mar 5;145(3):341–3.

11. Schutt PE, Kung S, Clark MM, Koball AM, Grothe KB. Comparing the Beck Depression Inventory-II (BDI-II) and Patient Health Questionnaire (PHQ-9) Depression Measures in an Outpatient Bariatric Clinic. OBES SURG. 2016 Jun 1;26(6):1274–8.

12. Weobong B, Weiss HA, Cameron IM, Kung S, Patel V, Hollon SD. Measuring depression severity in global mental health: comparing the PHQ-9 and the BDI-II. Wellcome Open Research. 2018 Dec 28;3:165–165.

13. Rydin AO, Milaneschi Y, Quax R, Li J, Bosch JA, Schoevers RA, et al. A network analysis of depressive symptoms and metabolomics. Psychological Medicine. 2023 Nov;53(15):7385–94.

14. Haslbeck JMB, Waldorp LJ. Structure estimation for mixed graphical models in high-dimensional data [Internet]. arXiv; 2015 [cited 2024 Aug 5]. Available from: http://arxiv.org/abs/1510.05677

15. Anderson MJ. Permutation tests for univariate or multivariate analysis of variance and regression. Can J Fish Aquat Sci. 2001 Mar;58(3):626–39.

16. Davey Smith G, Hemani G. Mendelian randomization: genetic anchors for causal inference in epidemiological studies. Human Molecular Genetics. 2014 Sep 15;23(R1):R89–98.

17. Smith GD, Ebrahim S. Mendelian Randomization: Genetic Variants as Instruments for Strengthening Causal Inference in Observational Studies. In: Biosocial Surveys [Internet]. National Academies Press (US); 2008 [cited 2025 Feb 17]. Available from: https://www.ncbi.nlm.nih.gov/books/NBK62433/

18. Kavousi M, Bos MM, Barnes HJ, Lino Cardenas CL, Wong D, Lu H, et al. Multi-ancestry genome-wide study identifies effector genes and druggable pathways for coronary artery calcification. Nat Genet. 2023 Oct;55(10):1651–64.

19. Aragam KG, Jiang T, Goel A, Kanoni S, Wolford BN, Atri DS, et al. Discovery and systematic characterization of risk variants and genes for coronary artery disease in over a million participants. Nat Genet. 2022 Dec;54(12):1803–15.

20. Franceschini N, Giambartolomei C, de Vries PS, Finan C, Bis JC, Huntley RP, et al. GWAS and colocalization analyses implicate carotid intima-media thickness and carotid plaque loci in cardiovascular outcomes. Nat Commun. 2018 Dec 3;9(1):5141.

21. Mishra A, Malik R, Hachiya T, Jürgenson T, Namba S, Posner DC, et al. Stroke genetics informs drug discovery and risk prediction across ancestries. Nature. 2022 Nov;611(7934):115–23.

22. Howard DM, Adams MJ, Clarke TK, Hafferty JD, Gibson J, Shirali M, et al. Genome-wide meta-analysis of depression identifies 102 independent variants and highlights the importance of the prefrontal brain regions. Nat Neurosci. 2019 Mar;22(3):343–52.

23. Karjalainen MK, Karthikeyan S, Oliver-Williams C, Sliz E, Allara E, Fung WT, et al. Genome-wide characterization of circulating metabolic biomarkers. Nature. 2024 Apr;628(8006):130–8.

24. Durbin RM, Altshuler D, Durbin RM, Abecasis GR, Bentley DR, Chakravarti A, et al. A map of human genome variation from population-scale sequencing. Nature. 2010 Oct;467(7319):1061–73.
